# Genome-Wide Characterization of the Phosphofructokinase Gene Family in *Arabidopsis thaliana* and Functional Analysis of AtPFK2 in Stress Tolerance

**DOI:** 10.3390/ijms26146828

**Published:** 2025-07-16

**Authors:** Siyu Liu, Jiheng Gou, Yunni Tang, Yunxiao Wei, Rui Zhang

**Affiliations:** Biotechnology Research Institute, Chinese Academy of Agricultural Sciences, Beijing 100081, China; 18622050625@163.com (S.L.); gouchenyu926@163.com (J.G.); rudongohye@163.com (Y.T.); weiyunxiao@caas.cn (Y.W.)

**Keywords:** phosphofructokinase (*PFK*) gene family, *Arabidopsis thaliana*, *AtPFK2*, stress, co-expression network analysis

## Abstract

The phosphofructokinase (*PFK*) gene family plays a pivotal role in glycolysis and energy metabolism in plants. This study aimed to systematically characterize the *PFK* gene family in *Arabidopsis thaliana* at the genome-wide level and to investigate the function of AtPFK2 (ATP-dependent phosphofructokinase 2) in response to salt and drought stress. Through bioinformatics analysis, 11 *AtPFK* genes were identified. Phylogenetic analysis revealed that these PFK genes can be classified into two subfamilies: PFK and PFP. Notably, *AtPFK2* possesses a unique structure, containing only a single intron, and its promoter is enriched with stress- and hormone-responsive elements, such as ABRE and MBS. T-DNA insertion mutants (*pfk2*) exhibited slightly shorter roots but slightly higher fresh weight under stress conditions, whereas *Arabidopsis* lines *AtPFK2*-overexpressing (OE-PFK2) showed increased stress sensitivity, with inhibited root and leaf growth, leaf wilting, reduced malondialdehyde and chlorophyll content, and enhanced accumulation of proline and soluble sugars. Weighted gene co-expression network analysis (WGCNA) identified 14 stress-related modules, from which six core genes—*LBD41*, *TRP3*, *PP2-A3*, *SAUR10*, *IAA6*, and *JAZ1*—were selected. These genes are involved in glycine metabolism and plant hormone signaling. The results of this study indicate that AtPFK2 mediates stress responses by regulating osmoregulatory substances and hormone signaling pathways, offering new insights into the mechanisms of stress resistance in crops.

## 1. Introduction

Glycolysis is a fundamental process in cellular respiration and energy metabolism, playing a crucial role under normal physiological conditions and directly contributing to the adaptive regulation of plant responses to abiotic stress. Alterations in the expression of glycolysis-related genes can significantly influence plant tolerance to stress [1,2]. For instance, glycolysis regulates energy supply, the synthesis of osmotic protectants (such as proline and sucrose), and the detoxification of reactive oxygen species (ROS) [3,4,5,6,7]. Within this pathway, phosphofructokinase (PFK) and pyruvate kinase (PK) act as key rate-limiting enzymes, controlling the early and final steps of glycolysis, respectively [8,9,10]. These enzymes ensure cellular energy through the regulation of carbon flux [11]. Notably, the activity of PFK is dynamically regulated under stress conditions, e.g., salt and drought stress can induce the expression of certain plant *PFK* genes, thereby enhancing carbohydrate accumulation [12,13,14]. Beyond the well-characterized ATP-dependent PFK (EC 2.7.1.11), plants, some prokaryotes, and primitive organisms also possess PPi-dependent PFK (PFP, EC 2.7.1.90), which utilizes pyrophosphate (PPi) as a cofactor [15].

The discovery of these enzymes dates back to 1975, when Latzko and Kelly [16] first identified the chloroplast and cytosolic isoforms of PFK in spinach. Since then, substantial progress has been made in studying various ATP-dependent PFK isoforms in plants [9]. For example, spinach cytosolic PFK can be activated by phosphate, whereas the chloroplast isoform is slightly inhibited by it [17,18,19,20,21]. In 1979, a PPi-dependent PFK was isolated from pineapple leaves, laying the groundwork for PFP research in plants [22,23].

PFK plays a central role in plant growth and development, especially in regulating the allocation and utilization of photosynthetic products. Carbohydrates produced through photosynthesis require precise regulation by PFK to be properly distributed to different tissues and organs, supporting energy and carbon needs for processes like growth, flowering, and fruiting [24]. Moreover, in response to abiotic stress, PFK may enhance plant adaptability through several pathways: (1) osmotic regulation, promoting the conversion of fructose-6-P into osmotic protectants such as sucrose to alleviate cellular dehydration; (2) energy supply, maintaining ATP production when mitochondrial function is compromised; (3) redox balance, providing NADPH through glycolytic bypasses to neutralize ROS [5,25,26,27]. Notably, PFP, by utilizing PPi rather than ATP to catalyze reactions, may provide crucial metabolic flexibility under energy-limited stress conditions [28,29,30].

The whole-genome sequencing of the model plant Arabidopsis thaliana has provided an ideal platform for gene function research [31,32]. In recent years, studies on the *PFK* gene family in Arabidopsis have included analyses of enzyme activity, subcellular localization, and spatiotemporal expression patterns [8]. It has been found that the in vitro enzyme activity of AtPFK2 and AtPFK5 is significantly lower than that of other isoenzymes [15], and AtPFK5 is regulated by plastid thioredoxin-f-dependent redox regulation. Although the redox sensitivity of AtPFK5 has been established, the precise function of AtPFK2 under stress conditions remains unclear [15]. In this study, the salt treatment concentration and duration were determined based on previous research findings and preliminary experiments conducted with different concentrations. Previous studies have commonly used various concentrations of sodium chloride (NaCl) to simulate salt stress in plants [33].

The Arabidopsis *PFK* gene family comprises several members, which may exhibit functional redundancy or specificity. Therefore, a thorough analysis of its genomic organization, evolutionary history, and regulatory mechanisms is essential for understanding its biological functions. Traditional experimental methods, such as single-gene cloning and phenotype analysis, are difficult to apply on a genome-wide scale for identifying all members of a gene family systematically [34]. Recently, bioinformatics approaches have emerged as powerful tools for studying gene family evolution, structure, and function [35,36,37]. In this study, bioinformatics techniques were employed to systematically explore the Arabidopsis PFK gene family, identifying all its members and analyzing their gene structures, conserved domains, phylogenetic relationships, and *cis*-regulatory elements in the promoter region. Combining genetic and physiological experiments, this study focused on investigating the response of AtPFK2 to drought and salt stress, confirming its role in enhancing plant stress tolerance.

## 2. Results

### 2.1. Identification of the Phosphofructokinase (PFK) Gene Family in Arabidopsis thaliana

We identified the entire *PFKs* gene family in the *Arabidopsis thaliana* genome using BLASTP1.4.0 and HMMER3.1 software, followed by domain analysis to validate the proteins’ identities. A total of 11 PFK proteins containing the PFK domain (PF00365) were found, including AtPFK1-AtPFK7, AtPFPA1, AtPFPA2, AtPFPB1, and AtPFPB2 (Table 1). Bioinformatics analysis revealed substantial diversity in the physicochemical properties of these proteins. The amino acid lengths of these 11 proteins ranged from 444 residues (AtPFK2) to 617 residues (AtPFPA2), corresponding to molecular weights ranging from 49.2 kDa (AtPFK2) to 67.2 kDa (AtPFPA2). The isoelectric point (pI) values varied widely, ranging from 5.44 (AtPFPB2) to 8.46 (AtPFK4), with 7 of the proteins having pI values between 6.0 and 7.0. Stability analysis of the proteins revealed instability indices ranging from 29.2 (AtPFK2) to 42.07 (AtPFPA2), indicating differences in their stability profiles within cellular environments. Hydrophobicity analysis showed that the aliphatic indices ranged from 83.95 for AtPFPB2 to 94.38 for AtPFPA1, while the grand average of hydropathicity (GRAVY) values ranged from −0.27 for AtPFPB2 to −0.076 for AtPFPA1. These differences suggest that the solubility and functional efficiency of the PFK proteins may be modulated by their distinct hydrophilic and hydrophobic characteristics (Table 1). Chromosomal localization analysis revealed that the PFK family members are distributed across chromosomes 1, 2, 4, and 5 in *A. thaliana*. This pattern of distribution may be linked to functional diversification and evolutionary adaptation within this enzyme family. Subcellular localization predictions indicated that the PFK proteins are primarily localized in the cytoplasm, chloroplasts, and mitochondria (Table 1), reflecting the proteins’ functional versatility in cellular metabolism.

### 2.2. Phylogenetic Analysis of PFK Proteins in Arabidopsis thaliana

**Figure 1:**
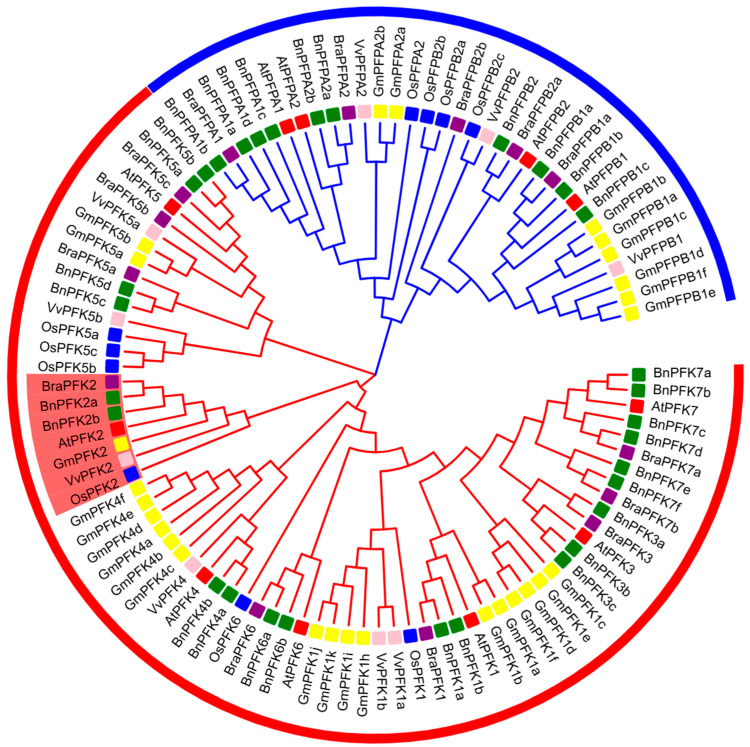
Phylogenetic relationships of the PFK proteins family in *Arabidopsis thaliana*. The phylogenetic tree was constructed using the Neighbor-Joining (NJ) method in MEGA11 software, with 1000 bootstrap replicates to assess support. In this tree, the PFK2 protein sequences from *Arabidopsis thaliana* were compared with PFK proteins from rice, grape, soybean, Chinese cabbage, and rapeseed. The outer rings, colored differently, represent the various species: red squares indicate *Arabidopsis*, blue squares represent rice, pink squares denote grape, yellow squares correspond to soybean, purple squares signify Chinese cabbage, and green squares represent rapeseed. This tree clearly illustrates the evolutionary relationships and functional divergence of the PFK protein family in *Arabidopsis thaliana* across different species.

To investigate the evolutionary relationships within the *PFK* gene family in *Arabidopsis thaliana*, we constructed a phylogenetic tree using the Neighbor-Joining (NJ) method. We performed a comparative analysis of PFK protein sequences from *A. thaliana*, *Oryza sativa* (rice), *Vitis vinifera* (grape), *Glycine max* (soybean), *Brassica rapa* (Chinese cabbage), and *Brassica napus* (rapeseed) through multiple sequence alignment. This provided the foundation for constructing the phylogenetic tree. The analysis revealed that the *A. thaliana* PFK proteins can be classified into two subfamilies: PFK and PFP. The PFK subfamily exhibits greater diversity in terms of members and functional variation, whereas the PFP subfamily is relatively conserved with fewer members. Comparative analysis showed that the PFK subfamily varies in size across species. In *O. sativa* (rice) and *V. vinifera* (grape), the subfamily contains six members, while in *A. thaliana*, there are seven members. In contrast, the PFK subfamily is notably expanded in *G. max* (soybean) with 20 members, *B. rapa* (Chinese cabbage) with nine members, and *B. napus* (rapeseed) with 21 members. The PFP subfamily remains relatively consistent in *A. thaliana*, *O. sativa*, and *V. vinifera*, but shows significant expansion in *B. napus* (10 genes) and *G. max* (8 genes) (Figure 1, Table 2).

Further examination of the PFK subfamilies revealed that the PFK1 family is the largest, with a total of 18 members (11 in soybean, 2 each in grape and rapeseed, and 1 each in rice, cabbage, and Arabidopsis). The PFK5 family consists of 15 members in total (4 in rapeseed, 3 each in rice and cabbage, 2 each in soybean and grape, and 1 in Arabidopsis). The PFK4 family contains 10 members (6 in soybean, 2 in rapeseed, 1 each in grape and Arabidopsis, and none in cabbage or rice). The PFK7 family has 9 members (6 in rapeseed, 2 in cabbage, 1 in Arabidopsis, and none in soybean, grape, or rice). The PFK2 family has 7 members in total (2 in rapeseed, and 1 each in soybean, grape, rice, cabbage, and Arabidopsis). The PFK3 and PFK6 families are relatively smaller, with 5 members each. PFK3 has 3 members in rapeseed, 1 in Arabidopsis and cabbage, and none in soybean, grape, or rice. PFK6 has 2 members in rapeseed, and 1 each in cabbage, rice, and Arabidopsis, with none in soybean or grape (Figure 1, Table 2). Notably, we found that the PFK2 protein family consists of a single member in most species, except for rapeseed, which contains two PFK2 proteins. This remarkable conservation of PFK2 across species suggests that it has undergone strong purifying selection during evolution, maintaining a relatively stable structure and function. The exceptional conservation of PFK2 highlights its potential as a core metabolic regulator in plants. These findings provide essential insights into the evolutionary history of the *Arabidopsis PFK* gene family and offer a valuable theoretical framework for understanding its role in plant metabolic regulation.

### 2.3. Motif and Gene Structure Analysis of the PFK Gene Family in Arabidopsis thaliana

**Figure 2:**
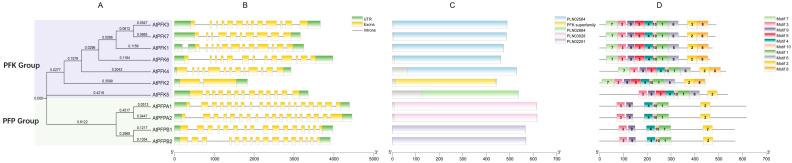
Features of the *PFK* genes in *Arabidopsis thaliana*. (**A**) A Neighbor-Joining (NJ) phylogenetic tree was constructed using MEGA11 software based on the full-length sequences of *Arabidopsis thaliana* PFK proteins. The tree’s reliability was assessed using 1000 bootstrap replicates. (**B**) Schematic representation of the gene structure of *Arabidopsis thaliana PFK* genes. Different colors correspond to various domains. Introns are shown as grey lines, exons as yellow rectangles, and untranslated regions (UTRs) as green rectangles. The scale bar represents 1 kb. (**C**) The domains of *Arabidopsis thaliana* PFK proteins. (**D**) Distribution of conserved motifs across *Arabidopsis thaliana* PFK proteins. Different colored boxes represent motifs 1–10, with a scale bar provided at the bottom.

Using the MEME suite, we conducted a conserved motif analysis of the *Arabidopsis thaliana* PFK protein sequences and identified 10 evolutionarily conserved motifs. These motifs displayed variable distribution patterns across the PFK members (Figure 2A,D). All members of the PFP subfamily contained 6 motifs and lacked motifs 5, 6, 7, and 8. In contrast, the PFK subfamily members, except for AtPFK5, which lacks motifs 7 and 8 and contains 8 motifs, all contained all 10 motifs. Notably, the C-terminal regions of the proteins exhibited higher sequence conservation, with motif 2 consistently localized in this region across most proteins. On the other hand, the N-terminal regions showed relatively lower conservation (Figure 2A,D). To further investigate the functional relevance of these motifs, we compared the 10 identified motifs with known domain databases (Pfam and InterPro). We found that, except for motifs 9 and 10, which did not match any sequences, the remaining motifs corresponded to known domains, including the phosphofructokinase A-type (PFKA) and phosphofructokinase superfamily (PFK_sf) (Table 3). Structural modeling using Phyre2 revealed varying proportions of disordered regions in the motifs, with motifs 3–5 showing the highest proportions. Motifs 7 and 8 also exhibited relatively high disordered regions (Figure S1). These results suggest that these motifs contribute to the protein’s functional regulation and structural flexibility, allowing it to adapt to different environmental conditions and participate in processes such as regulation, molecular interactions, and signal transduction. Further analysis showed that among all *Arabidopsis* PFK proteins, AtPFK1, AtPFK3, AtPFK4, AtPFK6, and AtPFK7 shared the same domain, PLN2564. AtPFK2 contained a unique PFK domain, while AtPFK5 contained the PLN02884 domain. AtPFPA1 and AtPFPA2 both contained the PLN03028 domain, and AtPFPB1 and AtPFPB2 shared the PLN02551 domain (Figure 2C). These findings indicate that, aside from maintaining core conserved domains, the *Arabidopsis* PFK proteins have evolved structural variations, reflecting functional specialization within the enzyme family. The subfamily-specific motif architectures likely contribute to biological functional diversity, with different motif combinations playing roles in specialized functions.

A comprehensive analysis of the coding sequence structures across the 11 *PFK* genes revealed variability in exon number within the coding regions (Figure 2B). Except for *AtPFK2*, which contains 2 exons, the other 10 *Arabidopsis PFK* genes consist of 10 to 19 exons and 9 to 18 introns per gene. *AtPFPA* contained 18 introns, while *AtPFK2* possessed only a single intron (Figure 2B). This suggests that *AtPFK2*, with its minimal exon–intron structure, may serve as a fundamental, conserved stress-responsive regulator, enabling rapid adaptation to environmental fluctuations. The observed structural polymorphism indicates divergent evolutionary trajectories among the *Arabidopsis PFK* genes. These variations in gene architecture likely reflect functional specialization, with distinct exon–intron organizations facilitating differential regulation in processes such as growth, development, and environmental stress responses. The structural fingerprints identified in this study provide critical insights into the mechanistic basis of functional diversification within the *PFK* gene family.

### 2.4. cis-Acting Element Analysis in the Promoter Regions of PFK Genes in Arabidopsis thaliana

**Figure 3:**
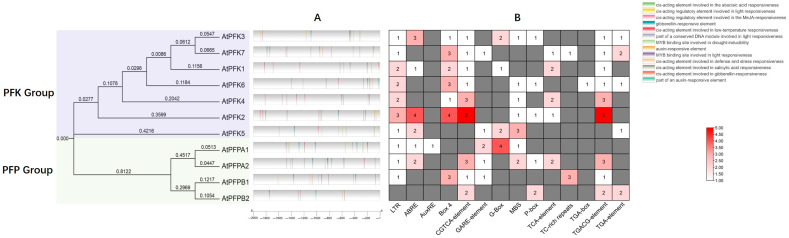
*cis*-acting elements in the promoters of *Arabidopsis thaliana PFK* genes. (**A**) Putative stress- and hormone-related *cis*-acting elements in the promoter regions of *Arabidopsis thaliana PFK* genes. These *cis*-acting elements were identified within the 2000-bp upstream promoter regions using the PlantCARE database. (**B**) Heatmap showing the distribution of putative stress-and hormone-related *cis*-acting elements. The names of the *cis*-acting elements are listed at the bottom of the figure, with numbers in the squares indicating the quantity of each element. Grey squares represent the absence of the element (quantity = 0).

Promoter analysis revealed that all *AtPFK* gene promoters contain multiple stress-responsive elements, but significant differences exist between family members (Figure 3A, Table 4). Notably, the promoter of *AtPFK2* is the most distinctive, containing 24 stress/hormone response elements, including 5 ABRE (ABA response elements) and 3 MBS (drought-responsive elements). In contrast, while *AtPFPB2* has fewer elements (8 in total) (Figure 3B, Table 4). To further investigate, we performed RT-qPCR analysis, *AtPFK2* exhibited significantly higher expression levels than *PFPB2* under salt stress, which contain fewer stress-responsive elements. A similar pattern was observed under drought stress (Figure S2), where the induction of *AtPFK2* was notably higher than that of other family members. Interestingly, despite the presence of a considerable number of stress-responsive elements in *PFPA1*, its expression remained lower than that of *AtPFK2*. These findings not only confirm that the quantity and composition of cis-acting elements directly influence the strength of stress responses but also highlight that *PFK* family members may form a hierarchical regulatory network in plant responses to environmental stresses. AtPFK2 acts as a core regulatory node responding to multiple stresses, while PFPA1 and others function as secondary responders involved in specific stress pathways. PFPB1/PFPB2, on the other hand, may primarily contribute to the regulation of basic metabolic processes. This cooperative model offers new insights into the metabolic regulatory mechanisms underlying plants’ complex adaptations to environmental changes.

### 2.5. AtPFK2 Acts as a Negative Regulator of Salt and Drought Stress Tolerance in Arabidopsis thaliana

To further investigate the functional role of AtPFK2, we screened and identified knockout mutants of *AtPFK2* (*AT5G47810*). Potential T-DNA insertion lines were identified from public databases and confirmed by RT-qPCR. We successfully identified the *PFK2* knockout mutant (line: SALK_088087C), which contains a T-DNA insertion in the exon (Figure S3A,B). To investigate the role of AtPFK2 in *Arabidopsis thaliana*’s response to adverse conditions, we first subjected the *pfk2* mutant (T-DNA) to salt and drought stress treatments. When grown on 1/2 MS medium supplemented with 100 mM NaCl, the roots of the *pfk2* mutant (T-DNA) were shorter than those of the wild-type (WT) plants (Figure S3C). However, the root systems were better developed, and the fresh weight increased. Similarly, when grown on 1/2 MS medium containing 300 mM mannitol, the roots of the *pfk2* mutant (T-DNA) were slightly shorter than those of the wild-type plants, but the root systems remained well developed, and the fresh weight also increased (Figure S3D,E).

**Figure 4:**
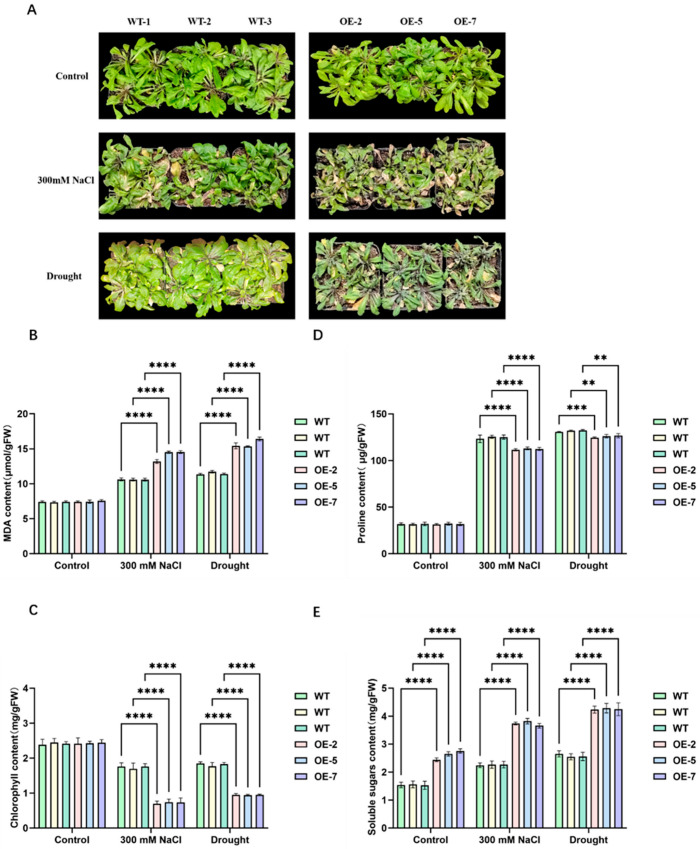
Functional analysis of the *AtPFK2* gene in *Arabidopsis thaliana* grown in soil under salt and drought stress. (**A**) Phenotypic comparison of wild-type (WT) and *AtPFK2*-overexpressing (OE) seedlings grown in normal soil (without NaCl) and salt-stressed soil (300 mM NaCl). Phenotypic observations were made 21 days after transplanting 13-day-old seedlings. (**B**–**E**) Levels of chlorophyll, malondialdehyde (MDA), proline, and soluble sugars in leaves. Data are expressed as the mean ± SD of three biological replicates. Asterisks indicate significant differences as determined by two-way ANOVA (** *p* < 0.01, *** *p* < 0.001, **** *p* < 0.0001).

To further explore the role of AtPFK2 in stress tolerance, we generated ten independent transgenic Arabidopsis lines overexpressing *AtPFK2*. From these, three representative overexpressing lines (OE-2, OE-5, and OE-7), which showed elevated transcript levels (Figure S4), were selected for detailed functional analysis. Three-week-old plants of both the *AtPFK2*-overexpressing (OE) and wild-type (WT) lines were subjected to either 300 mM NaCl irrigation or dehydration for seven days. Under normal watering conditions, there were no visible phenotypic differences between the OE and WT lines. However, after stress treatment, the differences became apparent. Wild-type plants exhibited moderate leaf wilting and partial chlorosis in the rosette leaves, whereas *AtPFK2*-overexpressing (OE) lines showed more severe wilting and extensive chlorosis (Figure 4A), suggesting that overexpression of *AtPFK2* increased stress sensitivity. Physiological analyses revealed that, compared to WT plants, the OE lines had significantly lower chlorophyll and proline contents under both salt and drought stresses (Figure 4C,D). In contrast, malondialdehyde and soluble sugar levels were elevated in the OE lines (Figure 4B,E). These findings indicate that *AtPFK2* overexpression compromises stress tolerance in *Arabidopsis*, making the transgenic plants more susceptible to both NaCl and drought stresses. This discovery not only enhances our understanding of AtPFK2’s role in stress adaptation and metabolic regulation but also provides a theoretical framework for the potential genetic improvement of crop stress tolerance through targeted manipulation of PFK2-mediated pathways.

### 2.6. Transcriptomic Analysis of AtPFK2 in Response to Salt and Drought Stresses and the Protein–Protein Interaction Network of Core Genes

To explore the regulatory mechanisms of AtPFK2 in stress adaptation, we performed RNA-Seq analysis on wild-type (WT), *AtPFK2*-overexpression (OE), and *pfk2* mutant (T-DNA) plants after 72 h of salt or drought treatment. This analysis aimed to identify differentially expressed genes (DEGs). Under salt stress, WT plants showed significant changes in 4304 genes (2557 upregulated and 1747 downregulated), while under drought stress, 3050 genes were affected (1534 upregulated and 1516 downregulated) (Figure S5A,B). In contrast, the gene expression differences between the wild-type (WT), *AtPFK2*-overexpression (OE), and *pfk2* mutant (T-DNA) plants were smaller.

**Figure 5:**
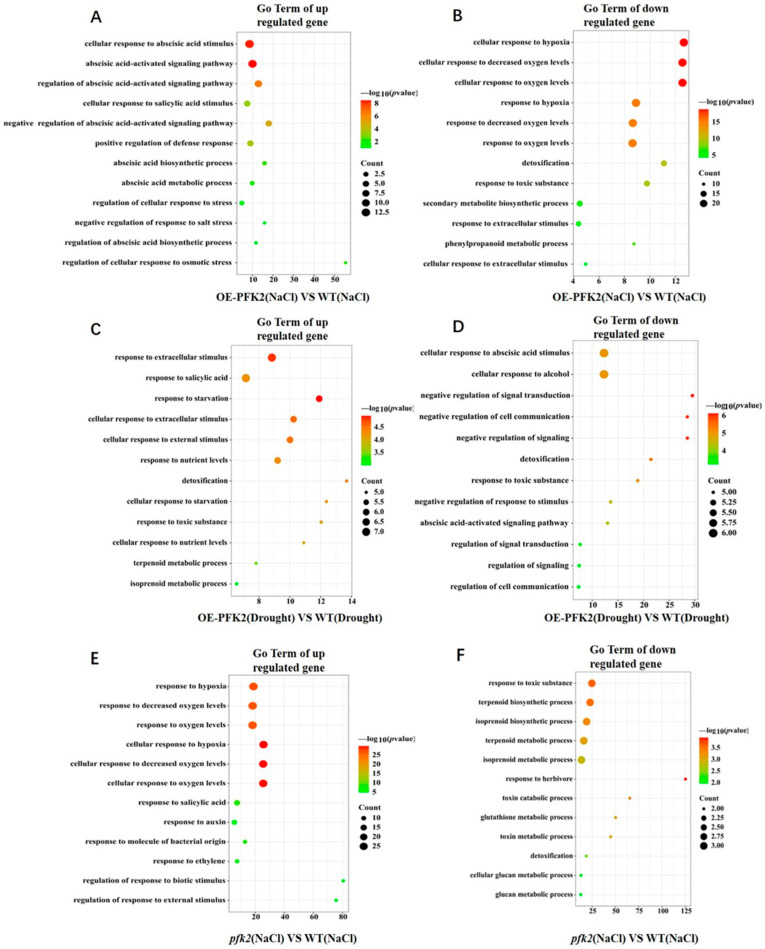

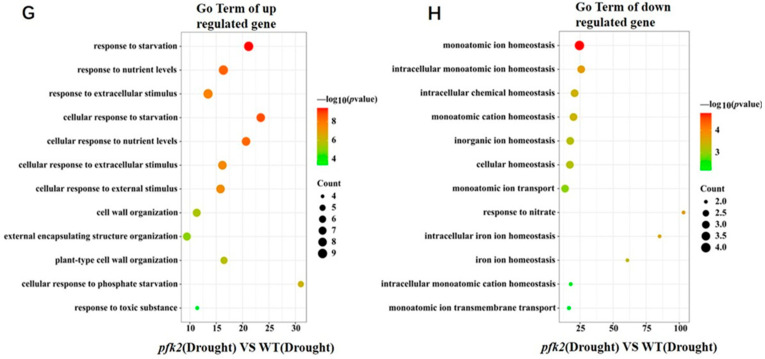
GO Enrichment analysis of core differentially expressed genes (DEGs) in wild-type (WT), *AtPFK2*-overexpression (OE), and *pfk2* mutant (T-DNA) lines under salt and drought stress. (**A**–**H**) show the twelve most significantly enriched biological processes for upregulated and downregulated genes. In these figures, the vertical axis represents various biological processes, and the horizontal axis displays the enrichment factor. The size of the dots corresponds to the number of enriched genes, while the color of the dots indicates the enrichment significance, represented by −log10(*p*-value).

Specifically, under salt stress, 304 genes were downregulated and 176 genes were upregulated in the *AtPFK2*-overexpression (OE) plants, while the *pfk2* mutant (T-DNA) plants showed 31 downregulated and 175 upregulated genes compared to the wild-type (WT) (Figure S5C,E). Gene Ontology (GO) analysis revealed that compared to the wild-type (WT), ABA pathway components were significantly upregulated in *AtPFK2* overexpression (OE), while genes involved in salicylic acid signaling were also activated (Figure 5A). Interestingly, downregulated genes were primarily associated with oxidative stress, toxin response, and various metabolic pathways (Figure 5B). Moreover, under salt stress, the majority of DEGs in wild-type (WT) plants compared to the *pfk2* mutant (T-DNA) were enriched in oxidative stress pathways, while salicylic acid and auxin signaling pathways were activated (Figure 5E). The downregulated genes were mainly related to toxic responses and secondary metabolite biosynthesis pathways (Figure 5F). Under drought stress, *AtPFK2*-overexpression (OE) plants exhibited 58 downregulated and 86 upregulated genes, while the *pfk2* mutant (T-DNA) plants had 21 downregulated and 75 upregulated genes compared to the wild-type (WT) (Figure S5D,F). Drought stress triggered distinct transcriptional responses. In *AtPFK2*-overexpression (OE) plants, extracellular stimulus perception and salicylic acid pathway genes were preferentially activated (Figure 5C), while ABA-related genes and those involved in ethanol response, negative regulation of cell signaling, and toxin response were suppressed (Figure 5D). In the *pfk2* mutant (T-DNA) plants, the upregulated genes were mostly enriched in responses to nutrient levels, extracellular stimulus perception, and hunger levels (Figure 5G), while genes related to intracellular monovalent ion homeostasis, cation homeostasis, and chemical homeostasis were suppressed (Figure 5H). These findings suggest that AtPFK2 orchestrates stress adaptation through pathway-specific modulation: (1) under salt stress, it primarily activates ABA-dependent signaling and toxic responses, while attenuating other stress responses; (2) during drought stress, AtPFK2 shifts its focus toward extracellular stimulus perception and the regulation of cellular ion homeostasis. This differential regulatory strategy positions AtPFK2 as a metabolic integrator, tailoring stress responses based on environmental challenges. These mechanistic insights advance our understanding of how central metabolic enzymes participate in stress signaling networks.

**Figure 6:**
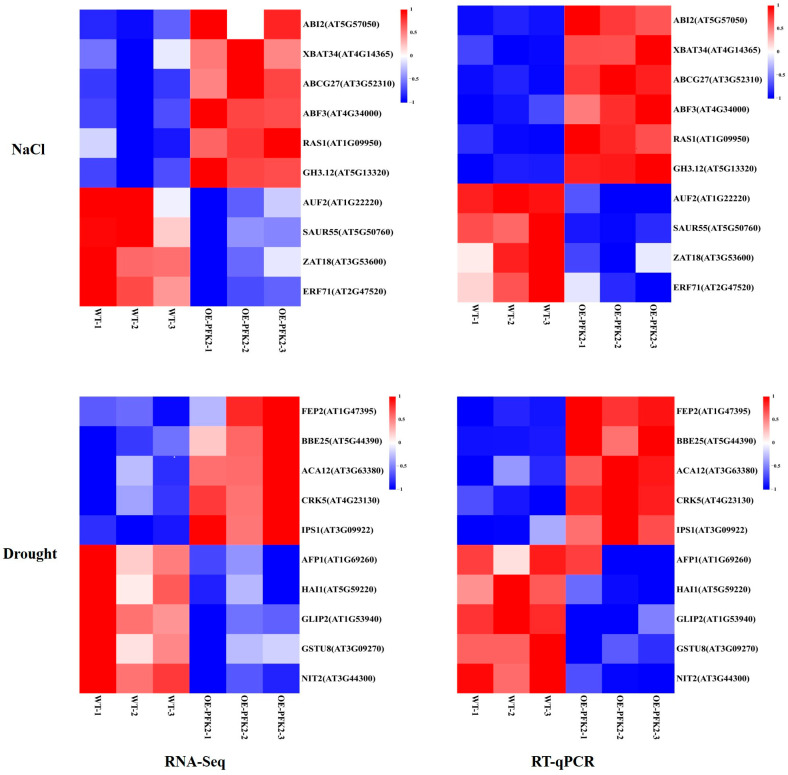
RT-qPCR validation of key genes in wild-type (WT) and *AtPFK2*-overexpression (OE) lines. The gene names and IDs are labeled on the right. The left panel displays the RNA-Seq results under salt and drought stress, while the right panel shows the RT-qPCR results under the same conditions. Both wild-type (WT) and *AtPFK2*-overexpression (OE) lines were analyzed in triplicate, with Actin used as the reference gene.

To validate the RNA-Seq findings on *Arabidopsis* responses to salt and drought stress, we performed quantitative real-time PCR (RT-qPCR) analysis on 20 differentially expressed genes (DEGs) that represent various functional categories (Figure 6). Under salt stress conditions, four ABA pathway-related genes (*ABCG27*, *RAS1*, *ABF3*, and *ABI2*) showed significant upregulation in the *AtPFK2*-overexpression (OE) lines compared to wild-type (WT) control plants. Simultaneously, genes related to salicylic acid signaling (*XBAT34*) and regulation of cellular responses to stress (*GH3.12*, excluding *AUF2* and *SAUR55*) were coordinately induced. However, two salt-responsive transcription factors (*ERF71* and *ZAT18*) were markedly downregulated. Drought stress elicited distinct expression patterns, two salicylic acid-responsive components (*BBE25* and *CRK5*) were highly upregulated in the *AtPFK2*-overexpression (OE) lines. Additionally, three extracellular stimulus sensors (*ACA12*, *IPS1*, and *FEP2*) were also upregulated, whereas five genes involved in toxin response, signaling regulation, and ABA signaling (*GSTU8*, *NIT2*, *GLIP2*, *AFP1*, and *HAI1*) were significantly suppressed. These RT-qPCR results are consistent with the RNA-Seq data, supporting the conclusion that AtPFK2 modulates a complex transcriptional network that affects (1) ABA-dependent signaling, (2) developmental reprogramming, and (3) extracellular signaling. The PCR analysis further confirms that overexpression of AtPFK2 compromises stress tolerance through the dysregulation of these critical physiological processes, highlighting its role as a metabolic regulator in plant–environment interactions.

**Figure 7:**
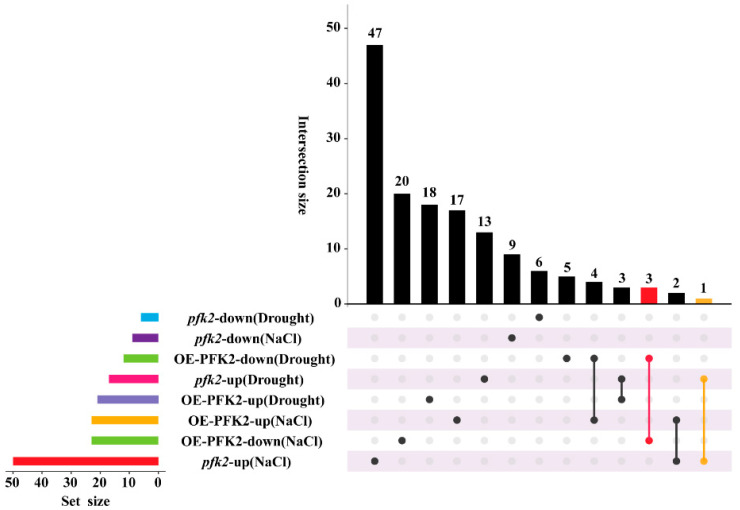
Analysis of upset plots for differentially expressed genes across different groups. In the main bar chart, different colors represent the number of samples in multiple combined groups, while the side bar chart displays the number of samples in each individual group. In the dot plot, each point on the horizontal axis corresponds to a gene set for each treatment, and the vertical axis represents the intersection of each gene set with others.

We also classified differentially expressed genes (DEGs) across eight comparative groups and identified distinct transcriptional reprogramming patterns under salt and drought stress conditions. In the *AtPFK2*-overexpression (OE) lines under NaCl stress, 17 upregulated DEGs were primarily enriched in ABA signaling pathways, while 20 downregulated DEGs were mainly related to oxidative stress responses. In contrast, 18 DEGs upregulated exclusively under drought stress were associated with extracellular stimulus perception and salicylic acid signaling pathways, and five DEGs were downregulated in both ABA signaling and alcohol response pathways (Figure 5A,D and Figure 7). KEGG pathway analysis revealed five significantly enriched metabolic and signaling pathways: “phytohormone signal transduction,” “glutathione metabolism,” “carbon metabolism,” “plant mapk signaling,” and “phenylpropanoid biosynthesis” (Figure S6A). In the *pfk2* mutant (T-DNA) under NaCl stress, 47 DEGs were upregulated, and 9 DEGs were downregulated, primarily enriched in oxidative stress responses and secondary metabolite biosynthesis pathways (Figure 5E,F and Figure 7). Under drought stress, 13 DEGs were upregulated, and 6 DEGs were downregulated in the mutant, with enrichment in extracellular stimulus and cellular ion homeostasis pathways (Figure 5G,H and Figure 7). In the *AtPFK2*-overexpression (OE) plants, three overlapping DEGs were downregulated under both salt and drought conditions, linked to responses to oxidative stress and ABA signaling pathways (Figure 5B,D and Figure 7). In contrast, in the mutant plants, one overlapping DEG was upregulated under both salt and drought conditions, associated with extracellular stimulus and hypoxia responses (Figure 5E,G and Figure 7). KEGG pathway analysis of the mutant plants identified several enriched pathways, including “plant–pathogen interaction,” “plant hormone signal transduction,” “secondary metabolite biosynthesis,” “glutathione metabolism,” “phenylpropanoid biosynthesis,” “carbon metabolism,” and “steroid biosynthesis” (Figure S6B).

**Figure 8:**
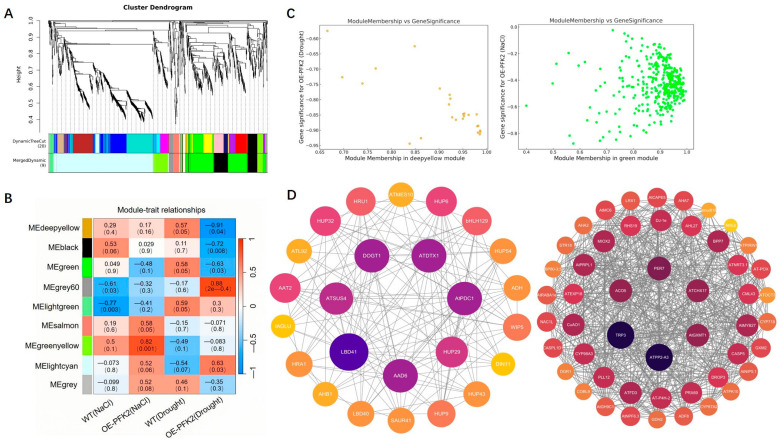
WGCNA analysis of AtPFK2-overexpression lines under salt and drought stress. (**A**) The gene dendrogram for the entire transcriptome profile was constructed using average linkage hierarchical clustering, where each branch represents a gene. The module assignments from the dynamic tree cut are shown in the module colors beneath the dendrogram. (**B**) Correlation between module feature genes and different genotypes. The color of each module corresponds to the color in panel (**A**). Genotypes for each module are displayed at the bottom of the module names. The correlation coefficient and *p*-value are shown in each cell. (**C**) Scatter plot of gene significance versus module membership for the deep yellow and green modules. (**D**) Co-expression network of the deep yellow and green module members. Each node represents a gene or protein, and the connections between nodes indicate their interactions. The strength or number of connections may reflect the intensity or type of interactions. The color of each node may represent different categories or groups, and the size of the node may indicate the importance or activity of a gene or protein, or the number of interactions with other genes/proteins.

**Figure 9:**
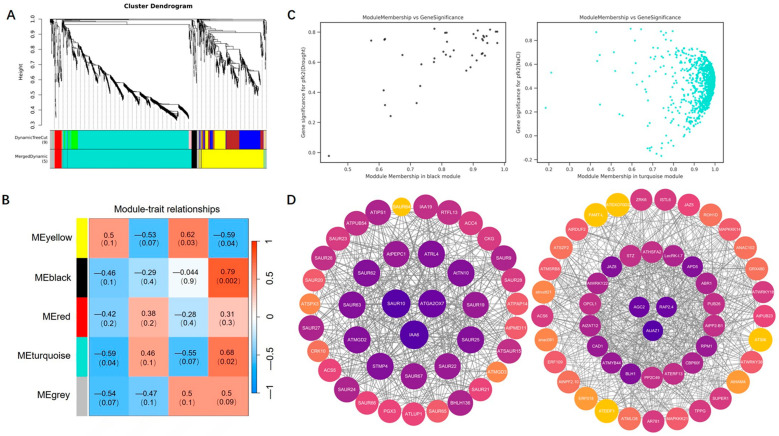
WGCNA analysis of the pfk2 mutant lines under salt and drought stress. (**A**) The gene dendrogram for the entire transcriptome profile was constructed using average linkage hierarchical clustering, where each branch represents a gene. The module assignments from the dynamic tree cut are shown in the module colors below the dendrogram. (**B**) Correlation between module feature genes and different genotypes. The color of each module corresponds to the color in panel (**A**). The gene numbers for each module are displayed at the bottom of the module names. The correlation coefficient and *p*-value are shown in each cell. (**C**) Scatter plot of gene significance versus module membership for the black and cyan modules. (**D**) Co-expression network of the black and cyan module members. Each node represents a gene or protein, and the connections between nodes indicate their interactions. The strength or quantity of the connections may reflect the intensity or type of interactions. The color of each node may represent different categories or groups, and the size of the node may indicate the importance or activity of a gene or protein, or the number of interactions with other genes/proteins.

To better understand the regulatory network of *AtPFK2* under salt and drought stress, we performed weighted gene co-expression network analysis (WGCNA) using expression datasets from four samples with three biological replicates. A total of 9 co-expression modules were identified in the *AtPFK2*-overexpression (OE) lines, and 5 modules were identified in the *pfk2* mutant (T-DNA) lines (similarity threshold > 0.25, gene expression threshold > 1), with each module labeled by a different color (Figure 8A and Figure 9A). Correlations between the modules and samples revealed that the deep yellow and green modules were negatively correlated with the *AtPFK2*-overexpression (OE) lines under drought and salt stress, respectively (r = −0.91, *p* = 4 × 10^−5^ and r = −0.48, *p* = 0.1) (Figure 8B). Conversely, the black and turquoise modules were positively correlated with the *pfk2* mutant (T-DNA) lines under drought and salt stress, respectively (r = 0.79, *p* = 0.002 and r = 0.46, *p* = 0.1), indicating their pivotal role in stress responses. Gene Ontology (GO) annotation of differentially expressed genes (DEGs) in the deep yellow and green modules revealed that the top 30 most enriched GO terms were associated with oxidative stress, hydrogen peroxide decomposition, cell wall modification, toxin catabolism, and nitrate transmembrane transport (Figure S7A,C). In contrast, the DEGs in the black and turquoise modules were enriched in terms related to auxin and brassinosteroid responses, temperature stress, salt stress, defense responses, and oxidative stress (Figure S7B,D). Scatter plots of gene significance (GS) and module membership (MM) for the deep yellow, green, black, and turquoise modules showed a high correlation between the two metrics, indicating that these modules are strongly linked to stress responses in both the *AtPFK2*-overexpression (OE) transgenic and *pfk2* mutant (T-DNA) lines (Figure 8C and Figure 9C). We further visualized gene interactions within the target modules using Cytoscape (3.10.3)software and identified the top 1, 2, or 3 genes with the highest degree as hub genes. In the deep yellow module, the hub gene was LBD41 (AT3G02550), while in the green module, the hub genes were *TRP3* (AT3G54640) and *PP2-A3* (AT2G26820) (Figure 8D). The hub genes identified in the black module were *SAUR10* (AT2G18010) and *IAA6* (AT1G52830), and in the turquoise module, the hub gene was *JAZ1* (AT1G19180) (Figure 9D). Functional annotation of these six hub genes revealed their involvement in glycine, serine, and threonine metabolism, as well as plant hormone signal transduction (Table 5). These findings suggest that *LBD41*, *TRP3*, *PP2-A3*, *SAUR10*, *IAA6*, and *JAZ1* may play critical roles in *Arabidopsis*’s response to salt and drought stress.

## 3. Discussion

Gene family investigations are crucial for understanding the mechanisms underlying plant growth, development, and responses to environmental stress [38]. As a central component of carbohydrate metabolism, the *Arabidopsis thaliana PFK* gene family has become a key focus in current research. However, the functional characterization and regulatory mechanisms of this family remain incompletely understood, necessitating further investigation. In this study, we utilized an integrated bioinformatic approaches to systematically analyze the *PFK* gene family in *Arabidopsis thaliana*. Phylogenetic analysis of *Arabidopsis* PFK proteins, in comparison with orthologs from five other plant species, revealed that *Arabidopsis* PFK proteins are divided into two distinct evolutionary branches (Figure 1). However, the expansion of the *PFK* family in *Arabidopsis* is relatively limited compared to species like *Brassica rapa*, *Brassica napus*, and *Glycine max*, which possess 9, 21, and 20 members in their *PFK* subfamilies, respectively. In contrast, *Arabidopsis* has only 7 members in its *PFK* subfamily. This reduced expansion could reflect unique evolutionary pressures, such as ecological competition or environmental shifts, leading to the loss of certain *PFK* family members in *Arabidopsis*. Unlike soybean and rice, where whole-genome duplication (WGD) events significantly contributed to gene family [39,40,41], *Arabidopsis* appears to have undergone fewer such large-scale genomic duplications. The remaining *PFK* genes in *Arabidopsis* have likely undergone functional specialization, addressing specific physiological needs, tissue-specific demands, developmental stage transitions [42], and environmental stress responses. Certain PFK isoforms, for instance, are preferentially expressed during seed germination to support energy-intensive growth [43], while others enhance glycolytic flux under stress conditions, providing metabolic flexibility [44,45,46,47]. This functional diversification, achieved through evolutionary refinement rather than gene family expansion, has likely conferred a competitive advantage within *Arabidopsis*’s ecological niche.

Our analysis of gene architecture, protein motifs, domain organization, and phylogenetic relationships revealed significant structural diversity within the *Arabidopsis* PFK family (Figure 2B). At the protein level, we identified ten conserved motifs (Figure 2D), with the AtPFK2 isoforms containing characteristic PFK domains essential for maintaining catalytic stability and allosteric regulation (Figure 2C) [48,49,50]. In contrast, PFP proteins possess distinct catalytic domains reflecting alternative enzymatic mechanisms [47,48,49]. Notably, we observed several disordered regions within these motifs, suggesting that they play crucial roles in maintaining the protein’s flexibility, enabling it to adapt with the environmental changes. Interestingly, motifs 9 and 10 did not match any known domains, which may indicate novel, yet-to-be-annotated functional regions, or portions of the protein that lack a clear function. While these auxiliary domains are not directly involved in catalysis, they are important for regulating tertiary structure and enhancing substrate binding affinity [51]. This structural variability underpins functional specialization within the PFK family, where differences in gene architecture and protein domain organization contribute to the regulation of various metabolic processes in plants.

This study reveals that the promoter regions of the Arabidopsis *PFK* gene family are enriched with cis-acting elements associated with methyl jasmonate (MeJA) and abscisic acid (ABA) (Figure 3A), offering new insights into the regulatory mechanisms of *PFK* genes in plant development and stress responses. Among the 11 *PFK* genes, *AtPFK2* stands out for its particularly rich collection of stress- and hormone-responsive elements (Figure 3B), especially those related to ABA and MeJA. This suggests that AtPFK2 may serve as a potential regulatory node for the integration of metabolic and stress signaling pathways. While the enrichment of ABA-responsive elements (ABRE) in the *AtPFK2* promoter holds theoretical significance, its actual functionality may be influenced by additional factors. Specifically, ABA-regulated sugar metabolic pathways may rely on the synergistic action of multiple genes [52,53,54,55], and the overexpression of a single *AtPFK2* gene alone is insufficient to activate the overall response. Likewise, the presence of MeJA-responsive elements did not directly correlate with enhanced defense phenotypes, suggesting that the function of AtPFK2 may be regulated by post-translational modifications or protein interaction networks [56,57]. Future studies should focus on exploring the regulation of AtPFK2’s protein activity, such as phosphorylation modifications, or its interactions with other metabolic enzymes to elucidate its true role under stress conditions. The results of this study demonstrate that overexpression of *AtPFK2* (phosphofructokinase 2) significantly impacts the metabolic balance and stress responses in plants. The observed decrease in proline and chlorophyll content may be attributed to an enhanced glycolytic flux, which directs carbon metabolism toward the synthesis of soluble sugars [58,59]. Additionally, the accumulation of soluble sugars could suppress the expression of genes involved in chlorophyll biosynthesis through feedback mechanisms [60] or induce oxidative damage to chlorophyll and thylakoid membranes via MDA-mediated damage [61], thereby impairing photosynthetic efficiency. The elevated MDA content further supports the accumulation of reactive oxygen species (ROS), which may be linked to electron transport chain leakage resulting from the increased glycolytic flux [62]. Importantly, *AtPFK2*-overexpressing plants exhibited higher expression levels of starch and sucrose metabolism-related genes, including BAMs, AMYs, SPSs, and SUSs, under salt and drought stress, consistent with the increased levels of soluble sugars (Figure S8). The upregulation of these genes likely enhances sugar breakdown and transport, thus providing additional energy and osmotic regulators to help the plant adapt to stress [63]. Previous studies have shown that soluble sugars, such as sucrose and trehalose, play a dual role in plant stress responses, serving as both osmotic protectants and ROS scavengers [64]. Consequently, AtPFK2 may enhance the plant’s ability to withstand abiotic stress by modulating the sugar metabolism network. However, the accumulation of MDA also suggests a potential risk of oxidative damage, indicating that AtPFK2-mediated metabolic reprogramming may act as a double-edged sword. Future studies should focus on optimizing the interplay between sugar metabolism and the antioxidant system to enhance plant stress tolerance while minimizing oxidative damage.

Plants adapt to stress conditions through the precise regulation of gene expression, which is a vital survival strategy [65,66]. Previous studies have indicated that certain *PFK* genes in *Arabidopsis* play crucial roles in responding to abiotic stresses, contributing to stress tolerance [67]. This study uncovers the complex regulatory role of AtPFK2 in plant responses to abiotic stress. Our findings suggest that AtPFK2 acts as a crucial regulatory node in sugar metabolism, modulating the plant’s stress adaptability by influencing the distribution of carbon flux. The observed metabolic disruptions and heightened stress sensitivity in *AtPFK2*-overexpression lines are consistent with previous studies that have reported an antagonistic relationship between sugar signaling and stress responses [68]. Notably, *AtPFK2* exhibits differential regulatory patterns in response to various stress types, which may be attributed to the activation of distinct signaling pathways under salt and drought stress conditions [69,70]. Gene expression analysis revealed that *AtPFK2* overexpression resulted in the downregulation of several stress-responsive genes, in line with reports suggesting that sugar signaling can suppress the expression of stress-related genes [71,72]. Conversely, the upregulation of specific genes in the mutant lines implies that plants may compensate for the loss of *AtPFK2* by activating alternative metabolic pathways, a phenomenon that highlights the plasticity of metabolic networks [73,74]. Noteworthy is the opposite regulation of certain genes under salt and drought stress, which may reflect the plant’s specific recognition mechanisms for distinct stress types [75]. These findings offer new perspectives on the interplay between plant metabolic reprogramming and stress adaptation. AtPFK2-mediated regulation of sugar metabolism likely balances the allocation of resources between growth and defense by influencing energy supply and signaling pathways. Future studies should further investigate the interactions between AtPFK2 and other stress signaling pathways, as well as its conservation across different plant species.

According to the WGCNA analysis, the salt and drought stress samples from the *AtPFK2*-overexpression lines show a strong negative correlation with the candidate core genes. Many of the genes with low expression in these samples are related to transporters, stress responses, transcription factors, and hormone signaling. In contrast, the salt and drought stress samples from the *pfk2* mutant exhibit a strong positive correlation with the candidate core genes, with many of the highly expressed genes also being involved in transporter functions, stress responses, transcription regulation, and hormone signaling. These categories of genes—transporters, stress-related genes, transcription factors, and hormone response genes—work synergistically to enhance plant stress resistance, aiding in the maintenance of growth, development, and survival under adverse environmental conditions [76,77,78,79]. KEGG enrichment analysis further supports this finding. The differentially expressed genes in the deep yellow and green modules are primarily enriched in pathways related to phenylpropanoid biosynthesis, plant hormone signaling, and amino acid metabolism. Meanwhile, the genes in the black and cyan modules are predominantly enriched in plant hormone signaling and plant–pathogen interactions (Figure S9). Notably, Vanholme et al. [80] observed that mutations in two key enzymes-*4CL1* and *CCR1*-in the phenylpropanoid pathway, which is involved in lignin biosynthesis, led to impaired lignin production. This disruption resulted in a 40% increase in water loss under drought conditions and reduced drought tolerance. Pauwels et al. [81] found that the absence of JA-induced activation of antioxidant genes, such as *GSTU24*, caused a threefold increase in MDA content under salt stress. Additionally, Pieterse et al. [82] demonstrated that pathogen-induced salicylic acid signaling activates *PR* genes via *NPR1*, enhancing PAL activity and promoting lignin synthesis, which in turn improves drought tolerance. In conclusion, the differential gene enrichment observed under salt and drought stress treatments is consistent with previous studies. AtPFK2 plays a critical role in enhancing Arabidopsis’ stress tolerance, potentially by integrating energy metabolism and stress response pathways, thereby improving plant adaptation to both salt and drought stress.

## 4. Materials and Methods

### 4.1. Genome-Wide Identification and Sequence Analysis

**Genome Data Acquisition:** The reference genome and gene annotation files for *Arabidopsis thaliana* were downloaded from the Phytozome13 database (https://phytozome.jgi.doe.gov/ (accessed on 5 March 2025)) and the Ensembl Plants database (https://plants.ensembl.org/).

***PFK* Gene Identification:** The *PFK* genes in *Arabidopsis thaliana*, soybean (*Glycine max*), rice (*Oryza sativa*), grape (*Vitis vinifera*), rapeseed (*Brassica napus*), and Chinese cabbage (*Brassica rapa*) were identified using a Hidden Markov Model (HMM) search. The PFK domain (PF00365) HMM profile was retrieved from the Pfam database (https://pfam.xfam.org/).

Domain Verification: Protein domains of AtPFK were validated using the SMART (http://smart.embl-heidelberg.de/ (accessed on 5 March 2025)) and CDD (https://www.ncbi.nlm.nih.gov/Structure/cdd/wrpsb.cgi (accessed on 5 March 2025)) online tools.

### 4.2. Prediction of Physiochemical Properties and Subcellular Localization

The biophysical and chemical properties of AtPFK proteins, including molecular weight, theoretical isoelectric point (pI), amino acid composition, atomic composition, extinction coefficient, estimated half-life, instability index, and aliphatic index, were analyzed using the Expasy ProtParam tool (https://web.expasy.org/protparam/ (accessed on 5 March 2025)). Subcellular localization of AtPFK proteins was predicted using the WoLF PSORT(v0.2) tool (https://wolfpsort.hgc.jp/ (accessed on 5 March 2025)).

### 4.3. Phylogenetic Analysis

The phylogenetic tree was constructed using the Neighbor-Joining (NJ) method in MEGA7 [83], with 1000 bootstrap replicates to assess the reliability of the phylogenetic relationships. The *PFK* genes from *Arabidopsis thaliana* were aligned with those from other species, including soybean, rice, grape, rapeseed, and Chinese cabbage. The phylogenetic relationships of these genes were used to classify them into distinct evolutionary groups.

### 4.4. Conserved Motif, Gene Structure, and cis-Element Analysis

The conserved motifs in the PFK proteins were predicted using the MEME Suite (https://meme-suite.org/meme/), setting the maximum number of motifs to 10, and visualized using TBtools (v2.310). Known domains were compared using Pfam, and motifs were modeled and analyzed with Phyre2.2 [84]. The intron–exon structure of *PFK* genes was examined using the Gene Structure Display Server 2.0 (http://gsds.gao-lab.org/index.php, (accessed on 5 March 2025)). The phylogenetic tree, conserved motifs, and exon–intron structures were visualized using TBtools (v2.310) [85] and Evolview (v2) [86]. The 2000 bp upstream DNA sequence of the ATG transcription start site of the *PFK* genes from the *Arabidopsis* genome was retrieved and analyzed for cis-elements using the PlantCARE website (http://bioinformatics.psb.ugent.be/webtools/plantcare/html/, (accessed on 5 March 2025)) [87]. *cis*-element quantities were statistically analyzed and visualized using TBtools (v2.310), and a heatmap was generated for further analysis.

### 4.5. Construction of AtPFK2-Overexpression Vector and Genetic Transformation in Arabidopsis

The full-length coding sequence of *AtPFK2* (TAIR ID:AT5G47810) was cloned into the pCAMBIA3301 vector, and PCR primers were designed based on the open reading frame (ORF) of the NCBI reference sequence NM_124155.3. The recombinant construct was transformed into *Agrobacterium tumefaciens* strain GV3101 (psoup) for transformation of *Arabidopsis thaliana* plants (wild type) [88]. The *Arabidopsis* seeds used were of the Columbia (Col-0) ecotype, stored in our laboratory. Homozygous T3 transgenic lines were selected using Basta (25 mg/L) (Coolaber, Beijing, Chian) for confirmation.

### 4.6. RNA-Seq and qRT-PCR Analysis

Wild-type (WT) and *AtPFK2*-overexpressing (OE) transgenic *Arabidopsis* seedlings were vertically grown on 1/2 MS (Murashige and Skoog) medium for 14 days, then transferred to soil for another 8 days. The plants were treated with 300 mM NaCl and water-withholding treatments for 7 days. Samples were collected on day 3 of treatment and stored at −80 °C for RNA extraction. Wild-type (WT) Arabidopsis seedlings were collected after 6 h of NaCl and drought treatment for RT-qPCR analysis of *PFK* and *PFPs* genes. Total RNA was extracted using the RNAprep Pure Plant Kit (TIANGEN, Beijing, China). RNA-Seq libraries were prepared using the TruSeqTM RNA Sample Prep Kit (Illumina, San Diego, CA, USA), and sequencing was performed on an Illumina platform. Sequences were aligned using HISAT2, transcript assembly was conducted with StringTie (v2.2.3), and differential expression analysis was performed using DESeq2. Transcriptional profiles of the *AtPFK2* gene were analyzed using heatmaps, bubble plots, and upset plots. Quantitative real-time PCR (qRT-PCR) was conducted using the Bio-Rad CFX Connect system (Bio-Rad, Hercules, CA, USA)with the *Arabidopsis* Actin gene as the reference. Each sample was analyzed in three technical replicates and three biological replicates.

### 4.7. Weighted Gene Co-Expression Network Analysis (WGCNA)

After constructing the weighted gene co-expression network analysis (WGCNA) and screening for differentially expressed genes (DEGs), we built a co-expression network of the DEGs using R software (4.4.2) and the WGCNA (v1.71) package, based on the gene expression profile matrix. To ensure a scale-free network distribution, the weight parameter power of the adjacency matrix was optimized. Power values were tested in the range of 1–30, and the correlation coefficients and average connectivity for networks corresponding to each power value were calculated. A higher correlation coefficient (with a maximum of 1) indicates that the network is closer to a scale-free distribution, while maintaining adequate gene connectivity. Therefore, we selected a power value that resulted in a sufficiently high correlation coefficient while preserving strong gene connectivity. Ultimately, we chose power = 30 to construct the neighbor connectivity matrix between genes and applied the topological overlap matrix similarity algorithm to transform this matrix into a topological overlap matrix. Based on the selected power of 30, a weighted co-expression network model was established, grouping 1627 genes and 1495 genes into 9 and 5 modules, respectively. The grey module (Grey) represents genes that cannot be assigned to any module and holds no significant reference value. These modules were then correlated with sample traits, and the correlation between each module and sample traits was calculated. After identifying significantly correlated modules, we performed GO and KEGG annotation and enrichment analyses on the genes within these modules. Finally, candidate core genes were visualized using Cytoscape 3.10.3 software, and the degree of each gene was calculated using the Centiscape 2.2 plugin to visualize the co-expression network. Node size was proportional to the degree, and genes with the highest degree were selected as hub genes.

### 4.8. Abiotic Stress Tolerance Assays

The wild-type (*Arabidopsis thaliana* Col-0) and mutant (*pfk2-TDNA*) lines were used in this experiment. Seeds were first sterilized with 70% ethanol for 1 min, followed by treatment with a 5% sodium hypochlorite solution for 10 min, and then washed five times with sterile water to remove any residual disinfectant. The sterilized seeds were evenly sown on 1/2 Murashige & Skoog (MS) medium (containing 3% sucrose and 0.8% agar, pH 5.8). The plates were cold-stratified at 4 °C in the dark for 2 days, then transferred to a growth chamber with a 16 h light/8 h dark cycle at 22 °C. After 7 days of cultivation, the seedlings were subjected to salt and simulated drought stress treatments. The mutant (*pfk2-TDNA*) lines were vertically placed on 1/2 MS medium plates containing different concentrations of NaCl (100 mM) and mannitol (300 mM), and after 7 days, the primary root lengths of the seedlings were measured using a ruler. At least four seedlings were measured per treatment, and the average root length was recorded. After 14 days of cultivation, the above-ground parts and root systems of the seedlings were collected, and surface moisture was removed using filter paper before weighing with an analytical balance to record the fresh weight. At least four seedlings were measured per treatment, and the average weight was recorded. For wild-type (WT) and *AtPFK2*-overexpressing (OE-PFK2) *Arabidopsis* seedlings, after vertical growth on 1/2 MS medium for 14 days, they were transplanted into soil and grown under greenhouse conditions at 22 °C with a 12 h light/12 h dark cycle for 8 days. Subsequently, the salt treatment group was irrigated with 2 L of a 300 mM NaCl solution per pot, and the control group was irrigated with the same volume of water. The drought treatment and control groups were subjected to water withholding, and the salt and drought stress treatments were maintained for one week. After treatment, the seedlings were photographed for records.

### 4.9. Determination of Proline, MDA, Chlorophyll, and Soluble Sugar Contents

To assess the effects of salt and drought treatments, seeds of wild-type (WT) and *AtPFK2*-overexpressing (OE) plants were grown on 1/2 MS medium for 14 days, then transplanted into soil for an additional 8 days of growth. The plants were then divided into two groups and treated under normal conditions with water, 300 mM NaCl, or water withholding for 1 week. Prolin, malondialdehyde (MDA), chlorophyll, and soluble sugar contents were measured using Proline (Pro) Content Assay Kit (Solar bio, BC0290, Beijing, China), Malondialdehyde (MDA) Content Assay Kit (Solar bio, BC0020), Plant Chlorophyll Content Assay Kit (Solar bio, BC0990), and Plant Soluble Sugar Content Assay Kit (Solar bio, BC0030), respectively [89].

### 4.10. Statistical Analysis

Statistical significance was determined using Student’s *t*-test. A *p*-value of <0.05 was considered statistically significant. All experiments were performed with at least three biological replicates and three technical replicates.

## 5. Conclusions

In this study, 11 *PFK* genes in *Arabidopsis* were identified and classified into two subfamilies: PFK and PFP. The *AtPFK2* gene, belonging to the PFK subfamily, possesses a simple gene structure with a single intron. A significant number of stress- and hormone-responsive *cis*-elements were identified in its promoter region, suggesting that AtPFK2 plays a critical role in regulating development and stress responses in *Arabidopsis*. Physiological analyses revealed that the AtPFK2 protein mitigates the effects of salt and drought stress. Additionally, co-expression network analysis through WGCNA uncovered co-expression modules with other proteins, with nine and five modules associated with the regulation of salt and drought stress, respectively. These findings provide a valuable foundation for further investigations into the biological functions of PFK proteins in plant growth, development, and stress adaptation.

## Figures and Tables

**Table 1:** Prediction of physicochemical properties and subcellular localization of AtPFK proteins.

Gene Name	Gene ID	Chromosome Localization	Number of Amino Acid	Molecular Weight	Theoretical pI	Instability Index	Aliphatic Index	Grand Average of Hydropathicity
*AtPFK1*	AT4G29220	Chr4	473	51,991.32	7.22	36.21	86.55	−0.20
*AtPFK2*	AT5G47810	Chr5	444	49,182.14	6.63	29.20	87.61	−0.19
*AtPFK3*	AT4G26270	Chr4	489	53,666.37	6.61	40.42	89.04	−0.19
*AtPFK4*	AT5G61580	Chr5	530	58,467.08	8.46	34.97	90.64	−0.17
*AtPFK5*	AT2G22480	Chr2	537	58,614.75	6.81	39.46	90.04	−0.14
*AtPFK6*	AT4G32840	Chr4	462	50,787.97	6.61	33.17	87.97	−0.17
*AtPFK7*	AT5G56630	Chr5	485	53,482.03	6.87	40.24	85.96	−0.28
*AtPFPA1*	AT1G20950	Chr1	614	67,119.18	6.53	40.62	94.38	−0.08
*AtPFPA2*	AT1G76550	Chr1	617	67,558.52	6.81	42.07	91.99	−0.12
*AtPFPB1*	AT1G12000	Chr1	566	61,459.46	5.80	37.17	88.07	−0.18
*AtPFPB2*	AT4G04040	Chr4	569	62,741.61	5.44	36.77	83.95	−0.27

**Table 2:** The gene names, corresponding gene IDs, and UniProt IDs of the *PFK* and *PFP* gene families in different species.

Species	Accession (Ensembl Plants)	Name	Accession (UniProt)
*Arabidopsis thaliana*	AT4g29220	AtPFK1	Q9M0F9
*Arabidopsis thaliana*	AT5g47810	AtPFK2	Q9FIK0
*Arabidopsis thaliana*	AT4g26270	AtPFK3	Q94AA4
*Arabidopsis thaliana*	AT5g61580	AtPFK4	Q9FKG3
*Arabidopsis thaliana*	AT2g22480	AtPFK5	Q8VYN6
*Arabidopsis thaliana*	AT4g32840	AtPFK6	Q9M076
*Arabidopsis thaliana*	AT5g56630	AtPFK7	Q9C5J7
*Arabidopsis thaliana*	AT1g20950	AtPFPA1	Q9SYP2
*Arabidopsis thaliana*	AT1g76550	AtPFPA2	Q9C9K3
*Arabidopsis thaliana*	AT1g12000	AtPFPB1	Q8W4M5
*Arabidopsis thaliana*	AT4g04040	AtPFPB2	F4JGR5
*Oryza sativa*	Os01g0191700	OsPFK1	Q0JPZ1
*Oryza sativa*	Os09g0479800	OsPFK2	Q652D3
*Oryza sativa*	Os10g0405600	OsPFK5a	A0A8J8YCT6
*Oryza sativa*	Os08g0439000	OsPFK5b	Q0J5F5
*Oryza sativa*	Os09g0415800	OsPFK5c	A0A0P0XM93
*Oryza sativa*	Os05g0524400	OsPFK6	Q65X97
*Oryza sativa*	Os06g0326400	OsPFPA2	B9FT08
*Oryza sativa*	Os09g0298100	OsPFPB2a	A0A0P0XL64
*Oryza sativa*	Os08g0345700	OsPFPB2b	Q84QT9
*Oryza sativa*	Os06g0247500	OsPFPB2c	A3BA88
*Glycine max*	GLYMA_06G088600	GmPFK1a	I1K9I0
*Glycine max*	GLYMA_06G088600	GmPFK1b	K7KU09
*Glycine max*	GLYMA_08G031700	GmPFK1c	I1KPV0
*Glycine max*	GLYMA_07G126400	GmPFK1d	I1KJS2
*Glycine max*	GLYMA_07G126400	GmPFK1e	I1KJS3
*Glycine max*	GLYMA_07G126400	GmPFK1f	I1KJS5
*Glycine max*	GLYMA_08G199800	GmPFK1h	I1KV07
*Glycine max*	GLYMA_13G353400	GmPFK1g	I1M5F6
*Glycine max*	GLYMA_13G353400	GmPFK1i	A0A0R0H5D7
*Glycine max*	GLYMA_15G020900	GmPFK1k	I1MCW1
*Glycine max*	GLYMA_15G020900	GmPFK1J	I1MCW0
*Glycine max*	GLYMA_07G269500	GmPFK2	I1KNN0
*Glycine max*	GLYMA_10G194300	GmPFK4a	A0A0R0HVU7
*Glycine max*	GLYMA_10G194300	GmPFK4b	A0A0R0HVV2
*Glycine max*	GLYMA_10G194300	GmPFK4c	I1LCJ1
*Glycine max*	GLYMA_10G194300	GmPFK4d	A0A0R0I3J4
*Glycine max*	GLYMA_10G194300	GmPFK4e	A0A0R0I3C2
*Glycine max*	GLYMA_10G194300	GmPFK4f	K7LKD1
*Glycine max*	GLYMA_08G280700	GmPFK5a	I1KX93
*Glycine max*	GLYMA_18G145500	GmPFK5b	I1N1M0
*Glycine max*	GLYMA_20G007400	GmPFPA2a	I1ND14
*Glycine max*	GLYMA_07G160500	GmPFPA2b	I1KKN7
*Glycine max*	GLYMA_09G007900	GmPFPB1a	I1KZW7
*Glycine max*	GLYMA_15G112300	GmPFPB1b	I1MFL2
*Glycine max*	GLYMA_15G112300	GmPFPB1c	A0A0R0FZ92
*Glycine max*	GLYMA_17G010100	GmPFPB1d	K7MJC5
*Glycine max*	GLYMA_07G263800	GmPFPB1e	I1KNH4
*Glycine max*	GLYMA_07G263800	GmPFPB1f	I1KNH5
*Brassica napus*	GSBRNA2T00091362001	BnPFK1a	A0A078IGT7
*Brassica napus*	GSBRNA2T00123079001	BnPFK1b	NO
*Brassica napus*	GSBRNA2T00001926001	BnPFK2a	A0A078FZE1
*Brassica napus*	GSBRNA2T00128839001	BnPFK2b	A0A816MMS9
*Brassica napus*	GSBRNA2T00082589001	BnPFK3a	A0A078JTU5
*Brassica napus*	GSBRNA2T00149578001	BnPFK3b	A0A816RM02
*Brassica napus*	GSBRNA2T00157466001	BnPFK3c	A0A816RM02
*Brassica napus*	GSBRNA2T00113526001	BnPFK4a	A0A816W200
*Brassica napus*	GSBRNA2T00021520001	BnPFK4b	A0A078GCL9
*Brassica napus*	GSBRNA2T00127444001	BnPFK5a	A0A816UWW4
*Brassica napus*	GSBRNA2T00078189001	BnPFK5b	A0A078FKS0
*Brassica napus*	GSBRNA2T00007196001	BnPFK5c	A0A078IRE3
*Brassica napus*	GSBRNA2T00150980001	BnPFK5d	A0A817AB30
*Brassica napus*	GSBRNA2T00121159001	BnPFK6a	NO
*Brassica napus*	GSBRNA2T00130627001	BnPFK6b	A0A816R9K6
*Brassica napus*	GSBRNA2T00073694001	BnPFK7a	A0A078HTA9
*Brassica napus*	GSBRNA2T00012921001	BnPFK7b	A0A078G2H5
*Brassica napus*	GSBRNA2T00052829001	BnPFK7c	A0A078H5N0
*Brassica napus*	GSBRNA2T00053002001	BnPFK7d	A0A078H7L8
*Brassica napus*	GSBRNA2T00060751001	BnPFK7e	A0A078FF53
*Brassica napus*	GSBRNA2T00148613001	BnPFK7f	A0A816V1T9
*Brassica napus*	GSBRNA2T00058643001	BnPFPA1a	A0A078JNQ8
*Brassica napus*	GSBRNA2T00059752001	BnPFPA1b	A0A078JHX5
*Brassica napus*	GSBRNA2T00102813001	BnPFPA1c	NO
*Brassica napus*	GSBRNA2T00055023001	BnPFPA1d	A0A078H4Z3
*Brassica napus*	GSBRNA2T00146592001	BnPFPA2a	A0A816ZCQ6
*Brassica napus*	GSBRNA2T00147797001	BnPFPA2b	NO
*Brassica napus*	GSBRNA2T00105468001	BnPFPB1a	NO
*Brassica napus*	GSBRNA2T00044584001	BnPFPB1b	A0A078GX24
*Brassica napus*	GSBRNA2T00057050001	BnPFPB1c	A0A078HAW1
*Brassica napus*	GSBRNA2T00157791001	BnPFPB2	NO
*Brassica rapa*	Bra011089	BraPFK1	M4D3N5
*Brassica rapa*	Bra024914	BraPFK2	M4E808
*Brassica rapa*	Bra026452	BraPFK3	A0A398AM83
*Brassica rapa*	Bra010637	BraPFK5a	M4D2D7
*Brassica rapa*	Bra030394	BraPFK5b	M4ENM4
*Brassica rapa*	Bra038519	BraPFK5c	M4FBQ3
*Brassica rapa*	Bra011387	BraPFK6	M4D4I3
*Brassica rapa*	Bra002801	BraPFK7a	M4CF19
*Brassica rapa*	Bra006864	BraPFK7b	M4CRM3
*Brassica rapa*	Bra025858	BraPFPA1	A0A397Z4U1
*Brassica rapa*	Bra015734	BraPFPA2	M4DGV8
*Brassica rapa*	Bra019733	BraPFPB1	M4DT90
*Brassica rapa*	Bra029482	BraPFPB2a	M4EL15
*Brassica rapa*	Bra016799	BraPFPB2b	M4DJX1
*Vitis vinifera*	Vitvi11g00237	VvPFK1a	A0A438DRZ9
*Vitis vinifera*	Vitvi04g00040	VvPFK1b	D7STJ8
*Vitis vinifera*	Vitvi10g00212	VvPFK2	A0A438D403
*Vitis vinifera*	Vitvi16g00381	VvPFK4	D7U799
*Vitis vinifera*	Vitvi07g01462	VvPFK5a	NO
*Vitis vinifera*	Vitvi14g01938	VvPFK5b	A0A438D7F2
*Vitis vinifera*	Vitvi18g00037	VvPFPA2	F6I6W5
*Vitis vinifera*	Vitvi10g00129	VvPFPB1	D7TR81
*Vitis vinifera*	Vitvi12g00427	VvPFPB2	D7TED0

**Table 3:** The alignment of various motifs in the *Arabidopsis thaliana PFK* gene family.

Sequence	Motif	E-Value	Entry Accession	Description
RRAGPRQKVYFEPDEVKACIVTCGGLCPGLNTVI	1	2.70 × 10^−17^	IPR035966	Phosphofructokinase superfamily
RRAGPRQKVYFEPDEVKACIVTCGGLCPGLNTVI	1	1.28 × 10^−15^	IPR035966	Phosphofructokinase superfamily
RRAGPRQKVYFEPDEVKACIVTCGGLCPGLNTVI	1	5.70 × 10^−26^	IPR050929	Phosphofructokinase type A
RRAGPRQKVYFEPDEVKACIVTCGGLCPGLNTVI	2	6.40 × 10^−18^	IPR050929	Phosphofructokinase type A
RRAGPRQKVYFEPDEVKACIVTCGGLCPGLNTVI	2	6.54 × 10^−12^	IPR035966	Phosphofructokinase superfamily
RRAGPRQKVYFEPDEVKACIVTCGGLCPGLNTVI	3	6.90 × 10^−16^	IPR050929	Phosphofructokinase type A
RRAGPRQKVYFEPDEVKACIVTCGGLCPGLNTVI	3	8.37 × 10^−10^	IPR035966	Phosphofructokinase superfamily
RRAGPRQKVYFEPDEVKACIVTCGGLCPGLNTVI	3	2.00 × 10^−6^	-	-
GGDGTQKGAAAIFEEIRRRKLKVAVVGIPKTIDNDI	4	1.70 × 10^−13^	-	-
GGDGTQKGAAAIFEEIRRRKLKVAVVGIPKTIDNDI	4	1.10 × 10^−14^	IPR050929	Phosphofructokinase type A
GGDGTQKGAAAIFEEIRRRKLKVAVVGIPKTIDNDI	4	3.79 × 10^−13^	IPR035966	Phosphofructokinase superfamily
GLVNGRHTYIPFNRITEKQNKVVITDRMWARLLSSTNQPSF	5	2.60 × 10^−12^	IPR050929	Phosphofructokinase type A
KVVNDIHKRGGTILGTSRGGHDTSKIVDSIQDRGINQVYII	6	2.20 × 10^−11^	-	-
KVVNDIHKRGGTILGTSRGGHDTSKIVDSIQDRGINQVYII	6	3.90 × 10^−16^	IPR050929	Phosphofructokinase type A
KVVNDIHKRGGTILGTSRGGHDTSKIVDSIQDRGINQVYII	6	1.70 × 10^−8^	IPR035966	Phosphofructokinase superfamily
KVVNDIHKRGGTILGTSRGGHDTSKIVDSIQDRGINQVYII	6	-	-	-
SPFYLEGKGGLFEFIEKRLKENGHMVIVIAEGAGQDLVAKSME	7	4.19 × 10^−5^	IPR035966	Phosphofructokinase superfamily
SPFYLEGKGGLFEFIEKRLKENGHMVIVIAEGAGQDLVAKSME	7	3.30 × 10^−6^	IPR035966	Phosphofructokinase superfamily
SPFYLEGKGGLFEFIEKRLKENGHMVIVIAEGAGQDLVAKSME	7	6.60 × 10^−15^	IPR050929	Phosphofructokinase type A
VPHLSDYLPDLPTYPNPLQDNPAYSVVKQYFVDADDTVPQKIVVHKDSPR	8	5.60 × 10^−14^	IPR050929	Phosphofructokinase type A

**Table 4:** Analysis of *cis*-acting elements in the −2000 bp promoter region of the *AtPFK* gene family in *Arabidopsis thaliana*.

Gene	ID	*cis*-Elements	Starting Position	Termination Position
*AtPFK1*	AT4G29220	LTR	−1828	−1822
*AtPFK1*	AT4G29220	CGTCA-motif	−1690	−1685
*AtPFK1*	AT4G29220	TGACG-motif	−1690	−1685
*AtPFK1*	AT4G29220	TATC-box	−1270	−1263
*AtPFK1*	AT4G29220	Box 4	−636	−630
*AtPFK1*	AT4G29220	Box 4	−576	−570
*AtPFK1*	AT4G29220	TCA-element	−476	−467
*AtPFK1*	AT4G29220	TCA-element	−207	−198
*AtPFK1*	AT4G29220	ABRE	−139	−134
*AtPFK1*	AT4G29220	G-box	−139	−133
*AtPFK1*	AT4G29220	LTR	−22	−16
*AtPFK2*	AT5G47810	LTR	−1896	−1890
*AtPFK2*	AT5G47810	LTR	−1854	−1848
*AtPFK2*	AT5G47810	TCA-element	−1811	−1802
*AtPFK2*	AT5G47810	CGTCA-motif	−1473	−1468
*AtPFK2*	AT5G47810	TGACG-motif	−1473	−1468
*AtPFK2*	AT5G47810	CGTCA-motif	−1470	−1465
*AtPFK2*	AT5G47810	TGACG-motif	−1470	−1465
*AtPFK2*	AT5G47810	CGTCA-motif	−970	−965
*AtPFK2*	AT5G47810	TGACG-motif	−970	−965
*AtPFK2*	AT5G47810	MBS	−949	−943
*AtPFK2*	AT5G47810	CGTCA-motif	−922	−917
*AtPFK2*	AT5G47810	TGACG-motif	−922	−917
*AtPFK2*	AT5G47810	P-box	−659	−652
*AtPFK2*	AT5G47810	ABRE	−262	−257
*AtPFK2*	AT5G47810	G-Box	−262	−256
*AtPFK2*	AT5G47810	LTR	−235	−229
*AtPFK2*	AT5G47810	CGTCA-motif	−85	−85
*AtPFK2*	AT5G47810	TGACG-motif	−85	−80
*AtPFK2*	AT5G47810	G-box	−84	−78
*AtPFK2*	AT5G47810	ABRE	−83	−78
*AtPFK2*	AT5G47810	G-box	−56	−46
*AtPFK2*	AT5G47810	ABRE	−55	−46
*AtPFK2*	AT5G47810	ABRE	−53	−48
*AtPFK2*	AT5G47810	G-box	−53	−47
*AtPFK3*	AT4G26270	ABRE	−1887	−1882
*AtPFK3*	AT4G26270	G-Box	−1887	−1881
*AtPFK3*	AT4G26270	CGTCA-motif	−1860	−1855
*AtPFK3*	AT4G26270	TGACG-motif	−1860	−1855
*AtPFK3*	AT4G26270	ABRE	−1165	−1160
*AtPFK3*	AT4G26270	G-Box	−1165	−1159
*AtPFK3*	AT4G26270	P-box	−1093	−1086
*AtPFK3*	AT4G26270	LTR	−486	−480
*AtPFK3*	AT4G26270	ABRE	−470	−463
*AtPFK3*	AT4G26270	Box 4	−464	−458
*AtPFK3*	AT4G26270	MBS	−423	−417
*AtPFK3*	AT4G26270	TGA-element	−321	−315
*AtPFK4*	AT5G61580	CGTCA-motif	−1818	−1813
*AtPFK4*	AT5G61580	TGACG-motif	−1818	−1813
*AtPFK4*	AT5G61580	Box 4	−1546	−1540
*AtPFK4*	AT5G61580	CGTCA-motif	−1476	−1471
*AtPFK4*	AT5G61580	TGACG-motif	−1476	−1471
*AtPFK4*	AT5G61580	LTR	−1320	−1314
*AtPFK4*	AT5G61580	LTR	−1191	−1185
*AtPFK4*	AT5G61580	TCA-element	−609	−600
*AtPFK4*	AT5G61580	MBS	−581	−575
*AtPFK4*	AT5G61580	CGTCA-motif	−190	−185
*AtPFK4*	AT5G61580	TGACG-motif	−190	−185
*AtPFK4*	AT5G61580	TCA-element	−173	−164
*AtPFK5*	AT2G22480	MBS	−1594	−1588
*AtPFK5*	AT2G22480	GARE-motif	−1357	−1350
*AtPFK5*	AT2G22480	LTR	−979	−973
*AtPFK5*	AT2G22480	ABRE	−921	−916
*AtPFK5*	AT2G22480	G-box	−921	−915
*AtPFK5*	AT2G22480	MBS	−657	−651
*AtPFK5*	AT2G22480	ABRE	−593	−588
*AtPFK5*	AT2G22480	G-box	−593	−587
*AtPFK5*	AT2G22480	MBS	−538	−532
*AtPFK5*	AT2G22480	TGA-element	−94	−88
*AtPFK6*	AT4G32840	Box 4	−1399	−1393
*AtPFK6*	AT4G32840	MBS	−1220	−1214
*AtPFK6*	AT4G32840	Box 4	−854	−848
*AtPFK6*	AT4G32840	LTR	−659	−653
*AtPFK6*	AT4G32840	Box 4	−265	−259
*AtPFK6*	AT4G32840	P-box	−198	−191
*AtPFK6*	AT4G32840	LTR	−164	−158
*AtPFK6*	AT4G32840	TGA-element	−123	−117
*AtPFK6*	AT4G32840	TGA-box	−28	−20
*AtPFK6*	AT4G32840	CGTCA-motif	−25	−20
*AtPFK6*	AT4G32840	TGACG-motif	−25	−20
*AtPFK7*	AT5G56630	GARE-motif	−1788	−1781
*AtPFK7*	AT5G56630	MRE	−1744	−1737
*AtPFK7*	AT5G56630	TC-rich repeats	−1500	−1491
*AtPFK7*	AT5G56630	Box 4	−1474	−1468
*AtPFK7*	AT5G56630	Box 4	−1095	−1089
*AtPFK7*	AT5G56630	LTR	−955	−949
*AtPFK7*	AT5G56630	TGA-element	−908	−902
*AtPFK7*	AT5G56630	TGA-element	−771	−765
*AtPFK7*	AT5G56630	Box 4	−427	−421
*AtPFK7*	AT5G56630	CGTCA-motif	−321	−316
*AtPFK7*	AT5G56630	TGACG-motif	−321	−316
*AtPFK7*	AT5G56630	TCA-element	−110	−101
*AtPFPα1*	AT1G20950	TATC-box	−1487	−1480
*AtPFPα1*	AT1G20950	G-box	−1454	−1448
*AtPFPα1*	AT1G20950	MBS	−1430	−1424
*AtPFPα1*	AT1G20950	GARE-motif	−1283	−1276
*AtPFPα1*	AT1G20950	LTR	−1195	−1189
*AtPFPα1*	AT1G20950	MRE	−1026	−1019
*AtPFPα1*	AT1G20950	G-box	−552	−546
*AtPFPα1*	AT1G20950	ATC-motif	−438	−430
*AtPFPα1*	AT1G20950	AuxRE	−371	−360
*AtPFPα1*	AT1G20950	G-box	−272	−264
*AtPFPα1*	AT1G20950	G-Box	−270	−264
*AtPFPα1*	AT1G20950	ABRE	−269	−264
*AtPFPα1*	AT1G20950	GARE-motif	−201	−194
*AtPFPα2*	AT1G76550	CGTCA-motif	−1477	−1472
*AtPFPα2*	AT1G76550	TGACG-motif	−1477	−1472
*AtPFPα2*	AT1G76550	MBS	−1415	−1409
*AtPFPα2*	AT1G76550	CGTCA-motif	−1387	−1382
*AtPFPα2*	AT1G76550	TGACG-motif	−1387	−1382
*AtPFPα2*	AT1G76550	ABRE	−1198	−1193
*AtPFPα2*	AT1G76550	P-box	−1066	−1059
*AtPFPα2*	AT1G76550	MBS	−870	−864
*AtPFPα2*	AT1G76550	ABRE	−834	−827
*AtPFPα2*	AT1G76550	GARE-motif	−828	−821
*AtPFPα2*	AT1G76550	TCA-element	−769	−760
*AtPFPα2*	AT1G76550	TCA-element	−575	−566
*AtPFPα2*	AT1G76550	LTR	−253	−247
*AtPFPα2*	AT1G76550	CGTCA-motif	−80	−75
*AtPFPα2*	AT1G76550	TGACG-motif	−80	−75
*AtPFPβ1*	AT1G12000	TC-rich repeats	−1613	−1604
*AtPFPβ1*	AT1G12000	MRE	−1369	−1362
*AtPFPβ1*	AT1G12000	TC-rich repeats	−1111	−1102
*AtPFPβ1*	AT1G12000	TC-rich repeats	−1075	−1066
*AtPFPβ1*	AT1G12000	Box 4	−973	−967
*AtPFPβ1*	AT1G12000	LTR	−925	−919
*AtPFPβ1*	AT1G12000	Box 4	−818	−812
*AtPFPβ1*	AT1G12000	Box 4	−421	−415
*AtPFPβ1*	AT1G12000	GARE-motif	−387	−380
*AtPFPβ1*	AT1G12000	CGTCA-motif	−75	−70
*AtPFPβ1*	AT1G12000	TGACG-motif	−75	−70
*AtPFPβ2*	AT4G04040	P-box	−1752	−1745
*AtPFPβ2*	AT4G04040	CGTCA-motif	−1295	−1290
*AtPFPβ2*	AT4G04040	TGACG-motif	−1295	−1290
*AtPFPβ2*	AT4G04040	CGTCA-motif	−1262	−1257
*AtPFPβ2*	AT4G04040	TGACG-motif	−1262	−1257
*AtPFPβ2*	AT4G04040	P-box	−1016	−1009
*AtPFPβ2*	AT4G04040	TGA-element	−599	−593
*AtPFPβ2*	AT4G04040	TGA-element	−566	−560

**Table 5:** Functional annotation of Hub genes in the key modules.

Module	Name	Gene ID	TFs Family	KEGG Pathway	Uniprot Annotation
Deepyellow	LBD41	AT3G02550	LBD	NO	LOB domain-containing protein 41
Green	TRP3	AT3G54640	NO	Glycine, serine and threonine metabolism	Indole-3-glycerol-phosphate lyase
Green	PP2-A3	AT2G26820	IAR	NO	Immune-associated nucleotide-binding protein 1
Black	SAUR10	AT2G18010	NO	Plant hormone signal transduction	Protein SMALL AUXIN UP-REGULATED RNA 10
Black	IAA6	AT1G52830	Aux/IAA	Plant hormone signal transduction	Auxin-responsive protein IAA6
Turquoise	JAZ1	AT1G19180	JAZ	Plant hormone signal transduction	Jasmonate ZIM domain-containing protein 1

## Data Availability

The raw transcriptome data generated in this study have not yet been submitted to the NCBI Sequence Read Archive. While data sharing is essential for validating and ensuring the reproducibility of results, the data have not been made publicly available as they are still undergoing further analysis. We acknowledge the importance of data sharing and will provide the data upon request for further validation or other research purposes.

## Supplementary Materials

The following supporting information can be downloaded at: https://www.mdpi.com/article/10.3390/ijms26146828/s1.
