# Supplementary material for: Genome-Wide Characterization of the Phosphofructokinase Gene Family in Arabidopsis thaliana and Functional Analysis of AtPFK2 in Stress Tolerance
Source: Int J Mol Sci. 2025 Jul 16;26(14):6828. doi: 10.3390/ijms26146828 (PMC12294930; doi:10.3390/ijms26146828)
Supplement: Supplementary file 1 [file ijms-26-06828-s001.zip › ijms-3641454-supplementary.pdf]

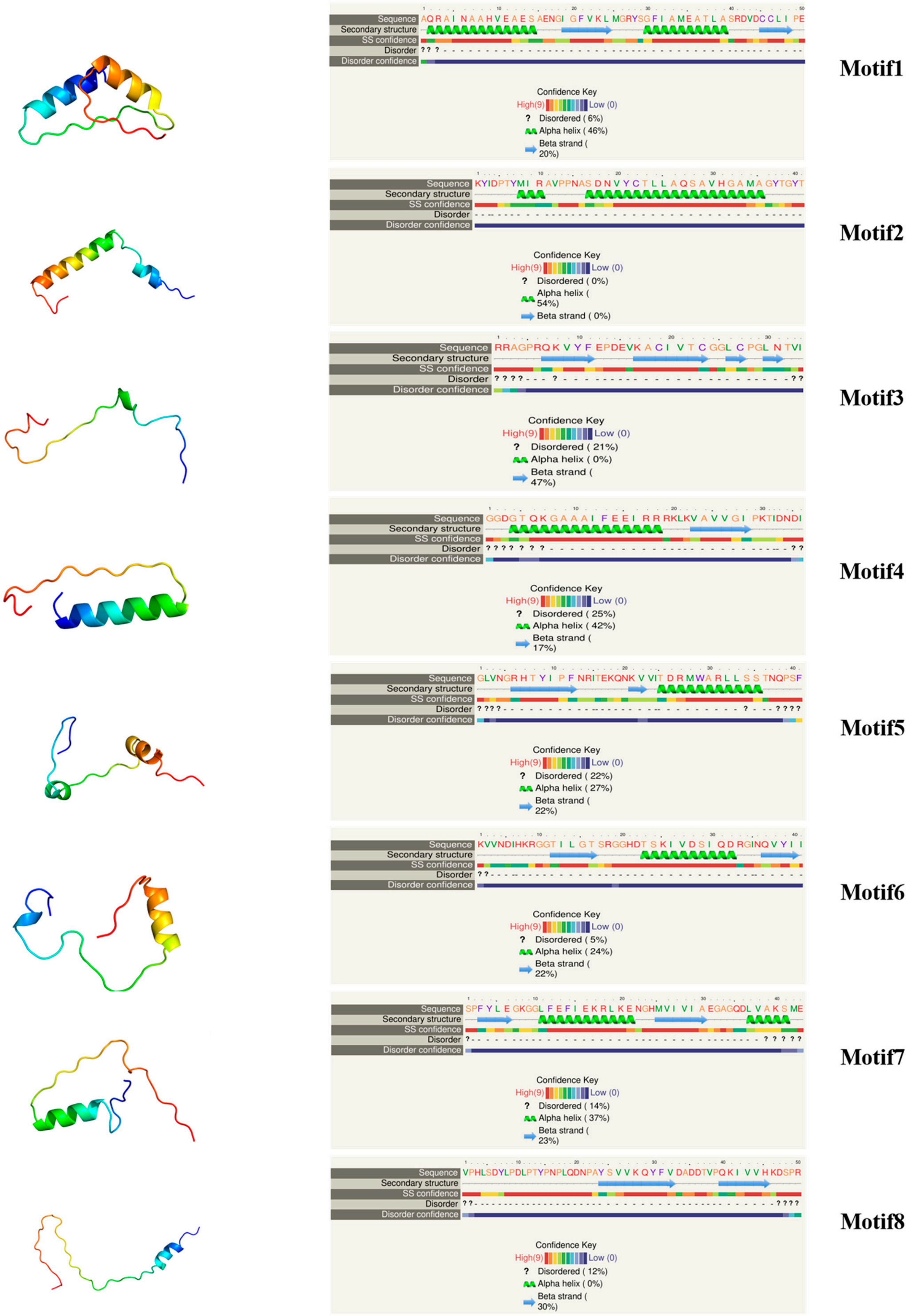

FigureS1. Structural analysis of conserved motifs using Phyre2. The 8 identified motifs were modeled and analyzed using Phyre2. The left panel shows the model diagrams of the 8 motifs, while the right panel presents the structural analysis of these models.

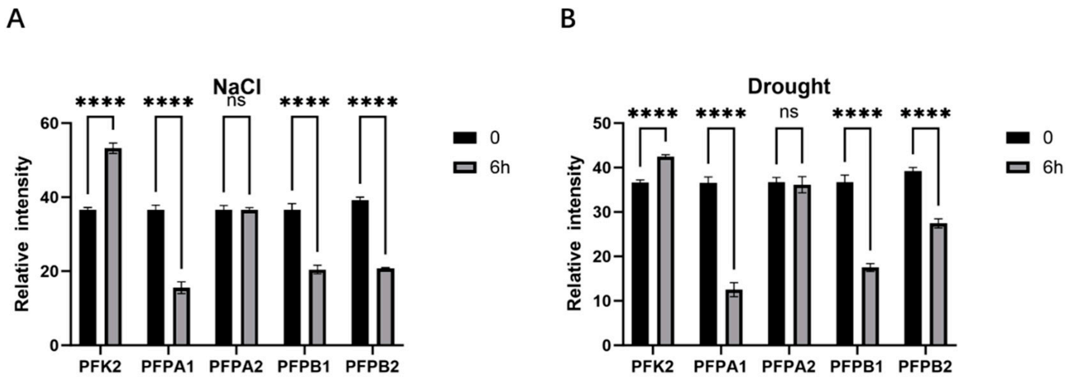

FigureS2. Analysis of the expression patterns of PFK gene family members under salt stress (NaCl) and drought stress conditions. (A) Relative expression levels of each gene under NaCl treatment. (B) Relative expression levels of each gene under drought stress conditions. * indicates a significant difference ($p < 0.05$).

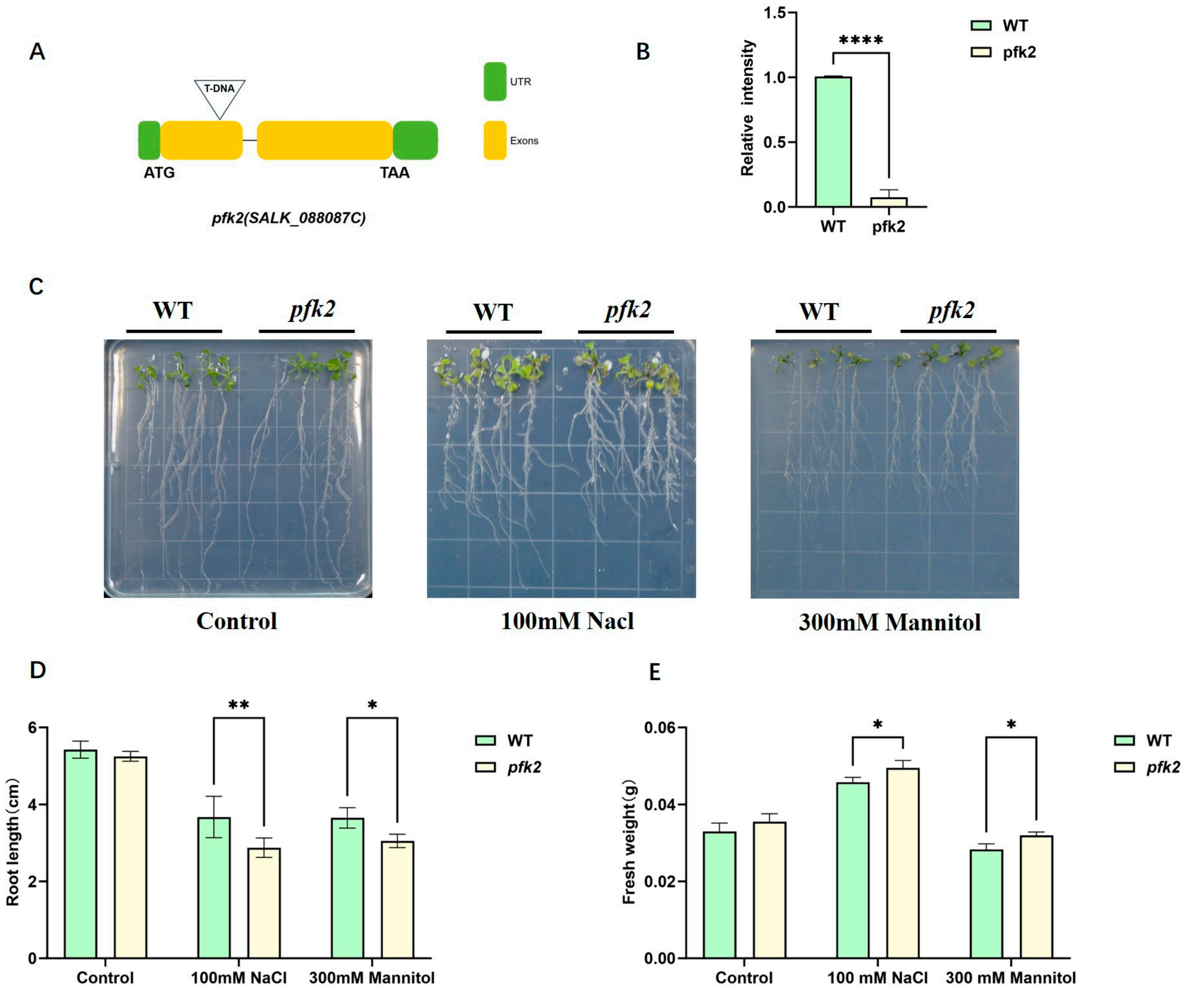

Figure S3. Comparison of wild-type (WT) and *pfk2* mutant (T-DNA) *Arabidopsis thaliana* under salt and simulated drought Stress. (A) and (B) The T-DNA insertion site in the *PFK2* gene (*AT5G47810*) was identified, and its gene expression levels were analyzed in the SALK_088087C T-DNA insertion line. (C) Phenotypic comparison of wild-type (WT) and *pfk2* mutant (T-DNA) *Arabidopsis thaliana* seedlings under normal conditions (1/2 MS medium), salt stress conditions (1/2 MS medium + 100 mM NaCl), and simulated drought stress conditions (1/2 MS medium + 300 mM Mannitol). (D) Effects of salt stress on the primary root length of wild-type (WT) and *pfk2* mutant (T-DNA) *Arabidopsis thaliana* seedlings. Data are presented as the mean ± SD of three biological replicates. * indicates a significant difference based on two- way ANOVA ($p < 0.05$). (E) Effects of salt and simulated drought stress on the fresh weight of wild-type (WT) and *pfk2* mutant (T-DNA) *Arabidopsis thaliana* seedlings. Data are presented as the mean ± SD of three biological replicates. * indicates a significant difference based on two-way ANOVA ($p < 0.05$).

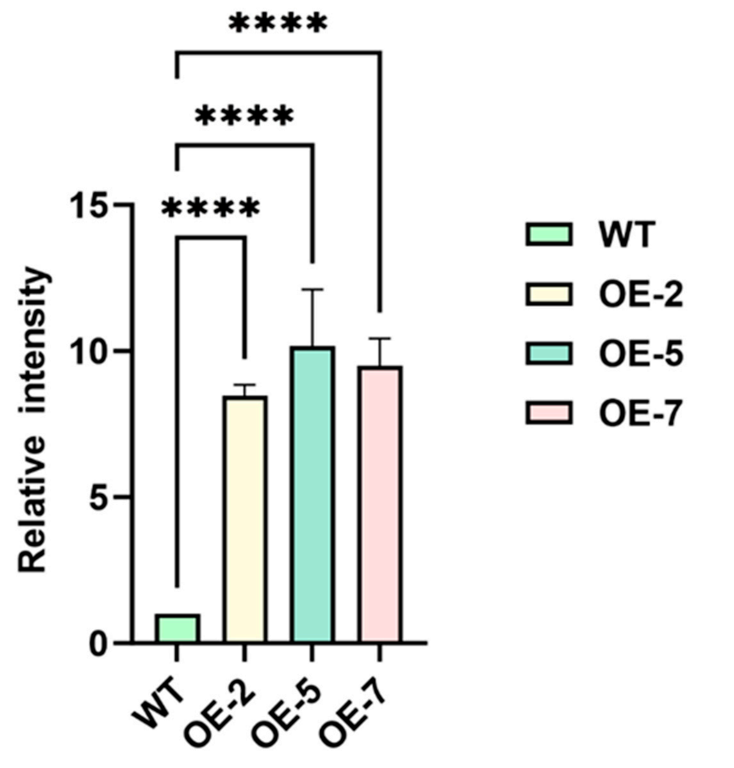

FigureS4. Expression analysis of *AtPFK2*. Relative expression of *AtPFK2* in transgenic Arabidopsis thaliana lines. The wild-type line Col-0 was used as a reference to calculate the relative expression in other lines. * indicates a significant difference ($p < 0.05$).

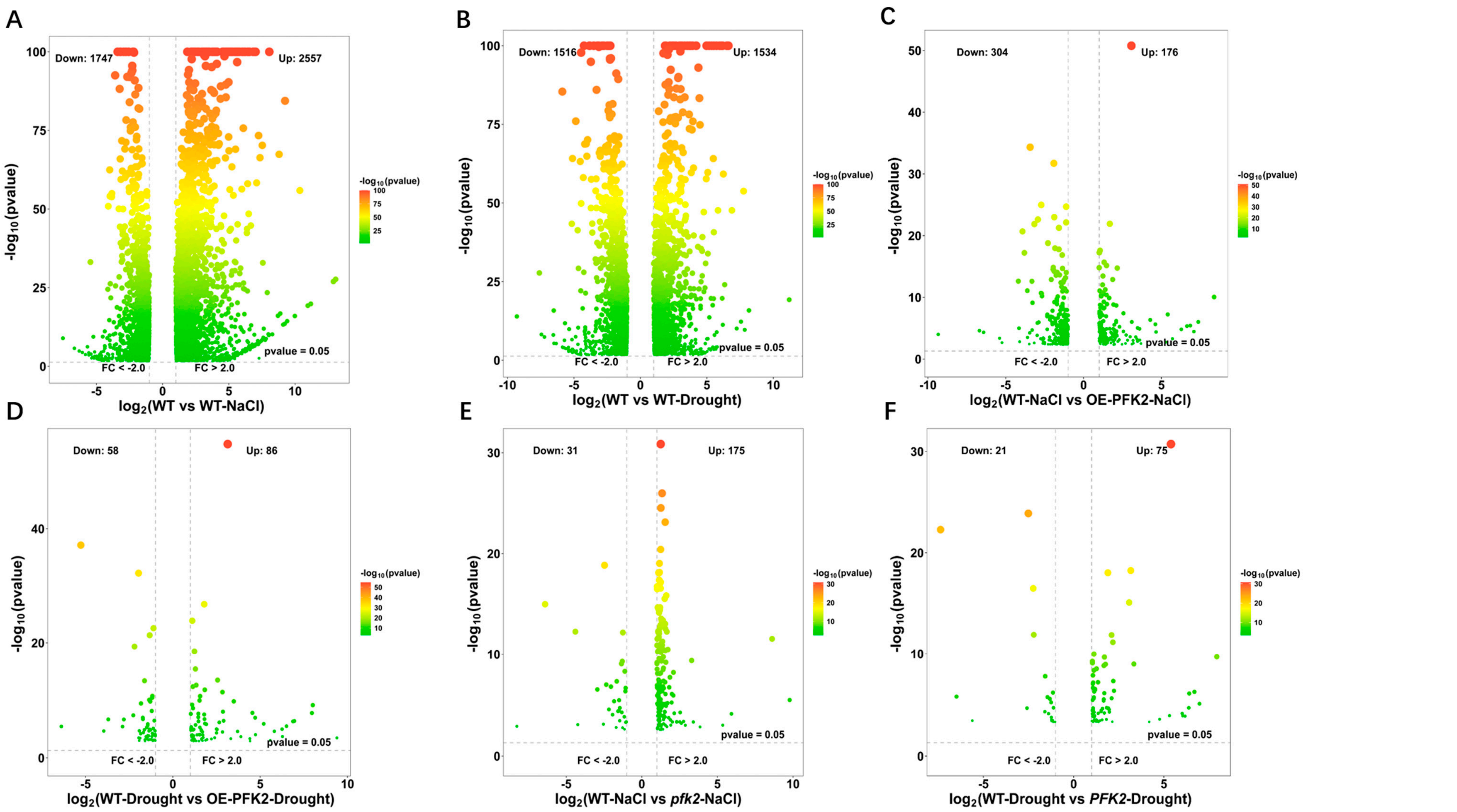

Figure S5. Volcano Plot of DEGs Mediated by *AtPFK2*. The vertical axis represents -log10(p-value), and the horizontal axis represents log2(FPKM). Genes on the right are upregulated, while those on the left are downregulated.

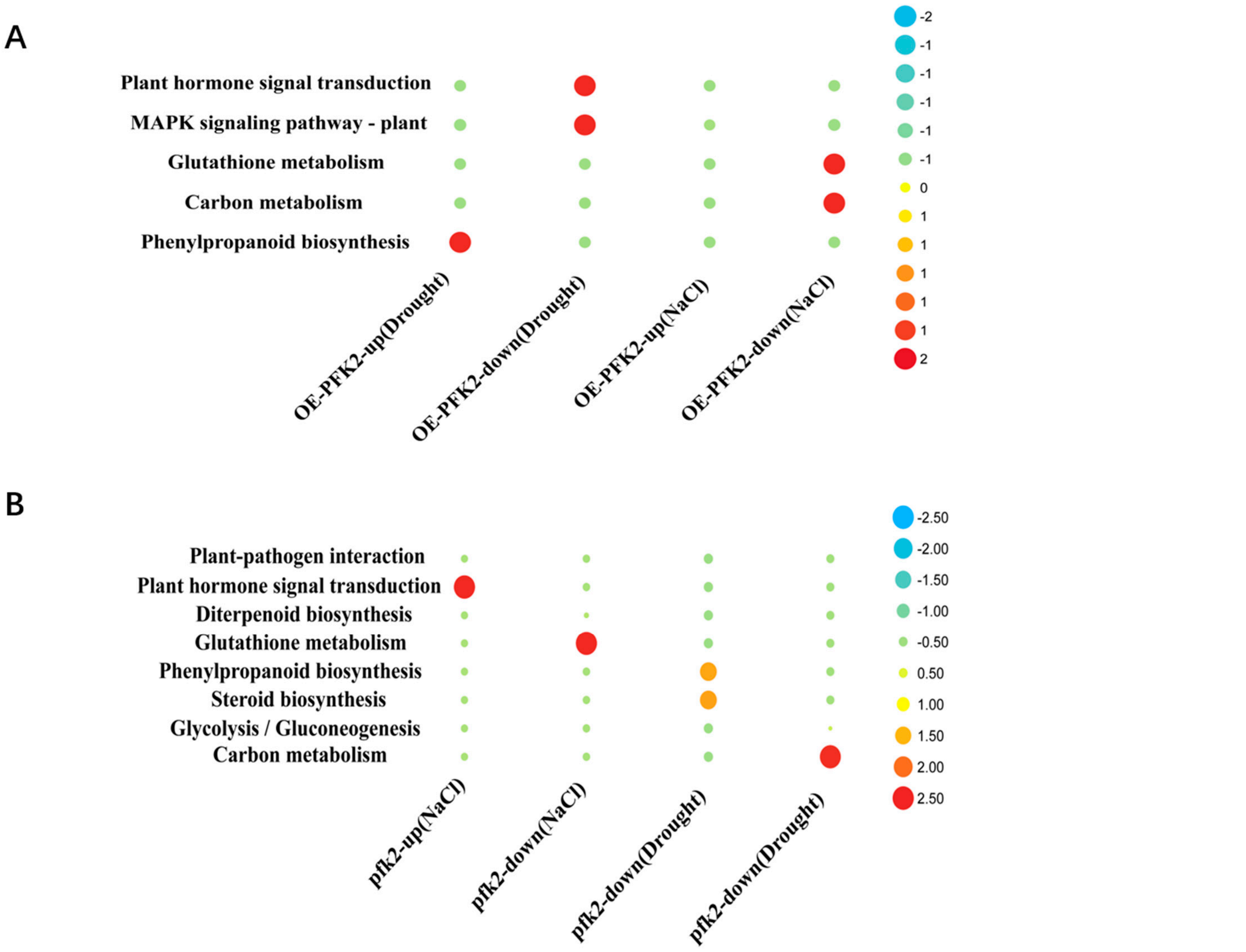

Figure S6. KEGG pathway enrichment analysis. (A) KEGG pathway enrichment analysis of *AtPFK2*-overexpression (OE) under salt and drought stress. (B) KEGG pathway enrichment analysis of *pfk2* mutant (T-DNA) under salt and drought stress. The bottom axis represents different experimental groups or conditions, while the left axis represents various KEGG pathways. Each circle represents a pathway, with the color and size of the circle indicating the level of enrichment under each condition.

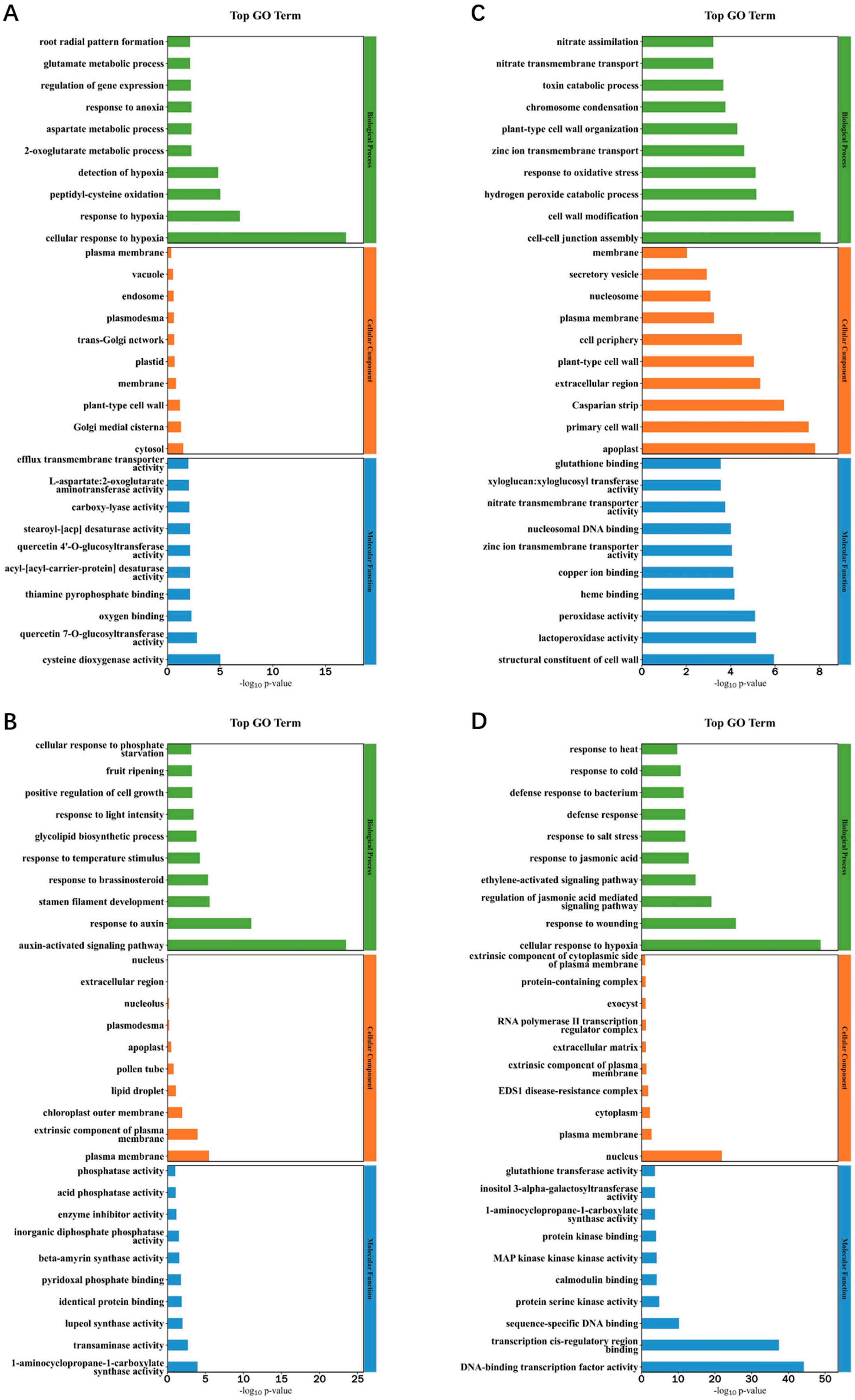

Figure S7. GO Enrichment analysis of candidate modules. (A) and (C) show the GO enrichment analysis of the deep yellow and green modules, respectively. (B) and (D) show the GO enrichment analysis of the black and turquoise modules, respectively. The horizontal axis represents -log(p-value), where a higher-log(p-value) indicates a stronger association between the GO term and specific changes (such as gene expression, diseases, etc.). The vertical axis represents different GO terms (Gene Ontology Terms), which describe the biological processes (BP), cellular components (CC), or molecular functions (MF) associated with the genes.

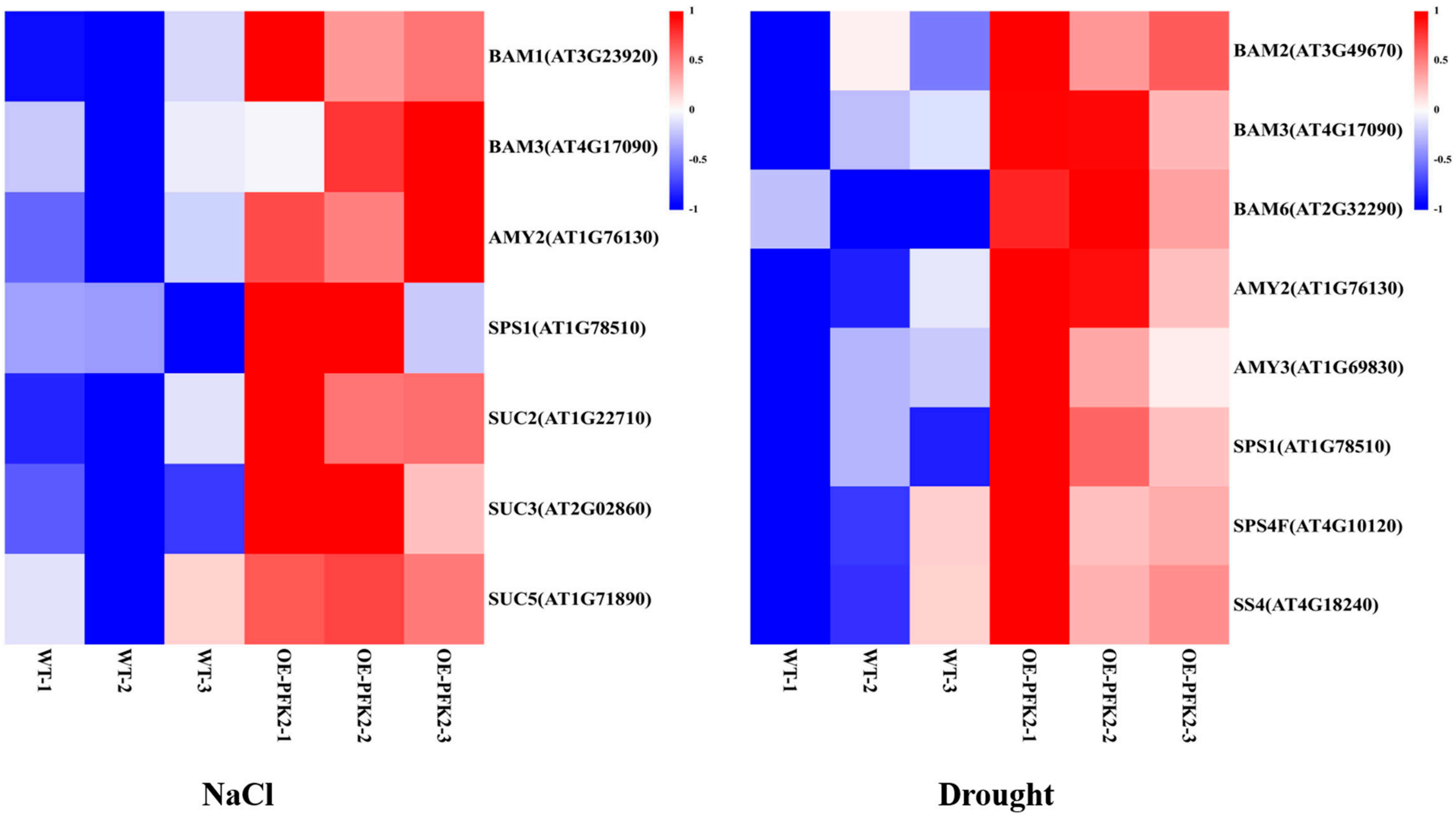

Figure S8. Heatmap of soluble sugar-related genes between wild-type (WT) and *AtPFK2*-Overexpressing (OE) Lines under salt and drought stress. The coordinates at the bottom of the heatmap indicate the names of each sample, while the right side of the heatmap displays the names and IDs of the corresponding genes.

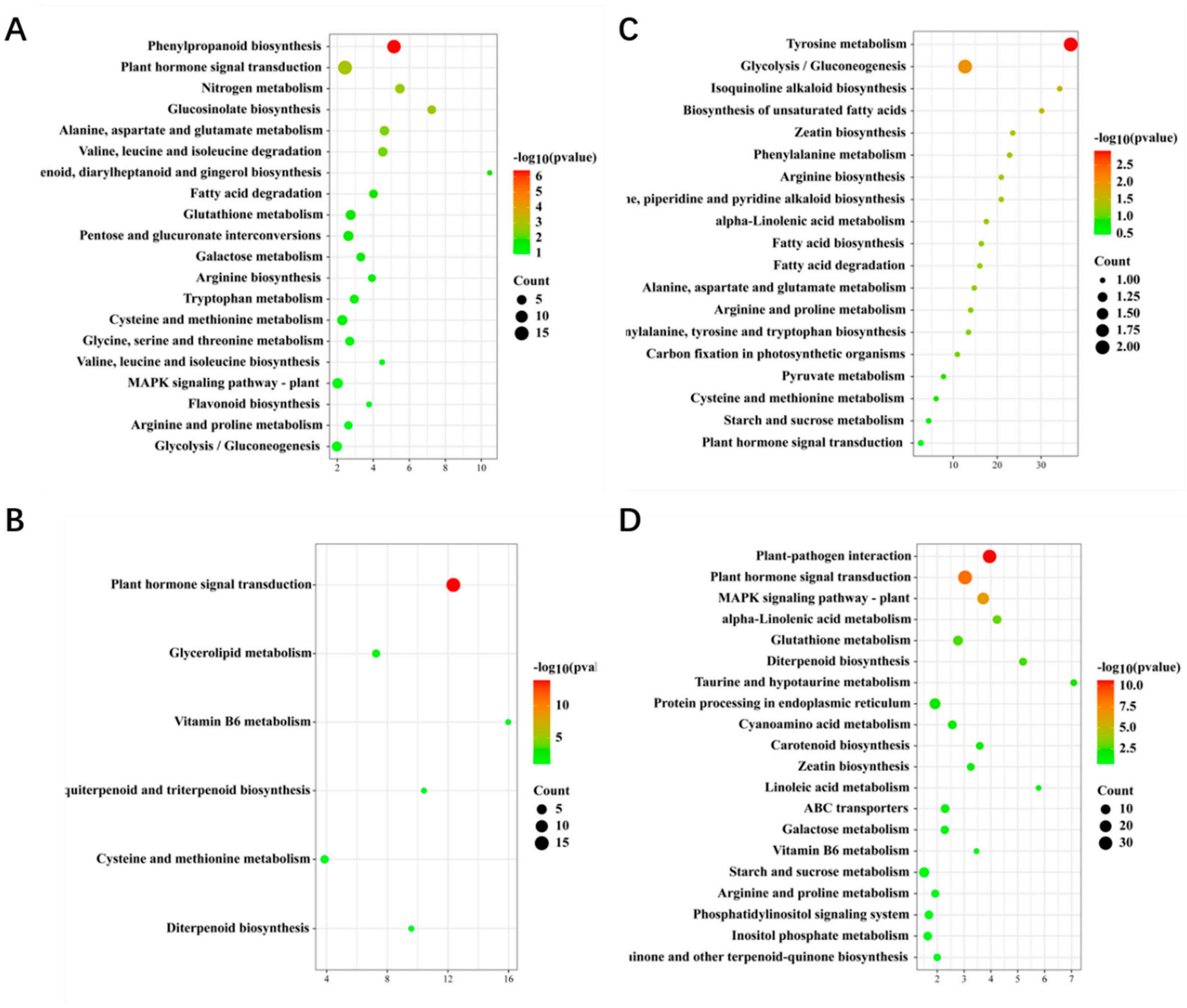

Figure S9. KEGG enrichment analysis of different modules in the co-expression network. (A) represents the deep yellow module, (B) represents the black module, (C) represents the green module, and (D) represents the turquoise module. In these figures, the vertical axis represents different biological processes, and the horizontal axis represents the enrichment factor. The size of the dots indicates the number of enriched genes, while the color of the dots reflects the enrichment significance (represented by -log10(p-value)).
